# Long and attenuated: comparative trends in the domestication of tree fruits

**DOI:** 10.1007/s00334-017-0659-2

**Published:** 2017-12-09

**Authors:** Dorian Q. Fuller

**Affiliations:** Institute of Archaeology, University College London, 31-34 Gordon Square, London, WC1H 0PY UK

**Keywords:** Arboriculture, Nuts, Unconscious selection, *Ziziphus*, *Prunus*, *Castanea*, *Olea*, *Phoenix*, Near East, China

## Abstract

This paper asks whether we can identify a recurrent domestication syndrome for tree crops (fruits, nuts) and track archaeologically the evolution of domestication of fruits from woody perennials. While archaeobotany has made major contributions to documenting the domestication process in cereals and other annual grains, long-lived perennials have received less comparative attention. Drawing on examples from across Eurasia, comparisons suggest a tendency for the larger domesticated fruits to contain seeds that are proportionally longer, thinner and with more pointed (acute to attenuated) apices. Therefore, although changes in flavour, such as increased sweetness, are not recoverable, seed metrics and shape provide an archaeological basis for tracking domestication episodes in fruits from woody perennials. Where available, metrical data suggest length increases, as well as size diversification over time, with examples drawn from the Jomon of Japan (*Castanea crenata*), Neolithic China (*Prunus persica*) and the later Neolithic of the Near East (*Olea europaea, Phoenix dactylifera*) to estimate rates of change. More limited data allow us to also compare Mesoamerica avocado (*Persea americana*) and western Pacific *Canarium* sp. nuts and *Spondias* sp. fruits. Data from modern Indian jujube (*Ziziphus mauritiana*) are also considered in relation to seed length:width trends in relation to fruit contents (flesh proportion, sugar content). Despite the long generation time in tree fruits, rates of change in their seeds are generally comparable to rates of phenotypic evolution in annual grain crops, suggesting that gradual evolution via unconscious selection played a key role in initial processes of tree domestication, and that this had begun in the later Neolithic once annual crops had been domesticated, in both west and east Asia.

**Electronic supplementary material:**

The online version of this article (10.1007/s00334-017-0659-2) contains supplementary material, which is available to authorized users.

## Introduction

The archaeobotanical documentation of plant domestication has made significant progress in the studying of the domestication of annual grain crops, including several cereals, pulses and some of the pseudo-cereals and oilseeds (Fuller et al. 2014). Recent insights from archaeobotany include recognition that the domestication process was protracted and that change to non-shattering (loss of wild-type seed dispersal) and grain size increase were both gradual over 2,000–4,000 years (Tanno and Willcox 2012; Fuller et al. 2012, 2014), but that domestication processes and rates of evolution show many parallels across crops and between geographical centres of origin (Fuller et al. 2014). Archaeobotany confirms parallel evolution towards a recurrent domestication syndrome in seed crops (Smith 2006; Fuller and Allaby 2009), including increasing grain size, which makes the recording of measurements on archaeobotanical specimens meaningful to studies of crop domestication. Archaeobotanists have debated the extent to which size criteria can be usefully used to determine the domestic status of early fruits (e.g. Runnels and Hansen 1986; Liphschitz and Bonani 2001; Liphschitz et al. 2013; Dighton et al. 2017) or whether it is mainly occurrence outside of the wild progenitor geographical range that indicates first cultivation (e.g. Weiss 2015). The present paper aims to explore the potential of large datasets of fruit and nut measurements to identify evolutionary processes of domestication, suggesting both a recognition of long term, gradual trends of size increase and the potential for such data to be compared in terms of evolutionary rates of change. While they draw upon the somewhat haphazard readily available data, the trends that appear suggest the potential for more systematic large scale studies of fruit size change and highlight apparent parallel evolution across several fruit domestications.

By contrast with the unconscious selection inferred for annual grain crop domestication, many fruits trees and vines have been suggested as involving more conscious selection processes as favoured individual plants were reproduced by vegetative propagation (Zohary and Spiegel-Roy 1975; cf.; Weiss 2015). It was further inferred that in West Asia most fruit domestications took place during the Chalcolithic or Bronze Age (Zohary and Spiegal-Roy 1975), millennia after annual grain crops (Goldschmidt 2013). Some archaeologists have connected fruit tree domestication with economic changes that involved “cash crop” production in parallel to growing specialization on animal secondary products, as societies became increasingly socially differentiated (Sherratt 1999). McCorriston (2009) flagged the importance of recognizing tree crops as indicators of stability of communities and land rights that emerged later than cereal agriculture. Nevertheless, it is possible that the beginnings of perennial fruit domestication took place in household gardens as a small component of mixed subsistence (Goldschmidt 2013), which would certainly fit with the evidence that figs were cultivated sometimes alongside pre-domesticated cereals (Kislev et al. 2006).

The present contribution explores the empirical archaeobotanical evidence for simple morphological change in a selection of tree fruits. In particular, fruit size and shape is linked to domestication processes. It asks whether chronological trends are evident in tree fruit (or nut) evidence that indicates gradual evolutionary processes, and therefore whether or not there is utility in collecting metrical data on archaeobotanical assemblages for analytical purposes other than merely describing finds. We can then ask to what extent these resemble or differ from those in annual seed crops, in the nature of change, the extent of change or the rate of change. Can we identify recurrent features that might be classified as a domestication syndrome for long-lived perennial fruit crops and how does this compare to the better documented domestication syndrome of annual seed crops (*sensu* Hammer 1984; Fuller 2007)? Goldschmidt (2013) argued that increasing productivity, reduction of juvenile period and reduced inter-annual variability in yield would all have been selected for in tree fruit domestication, although this cannot be directly determined archaeologically, except for the potential yield value per fruit, i.e. fruit size.

**Fig. 1:**
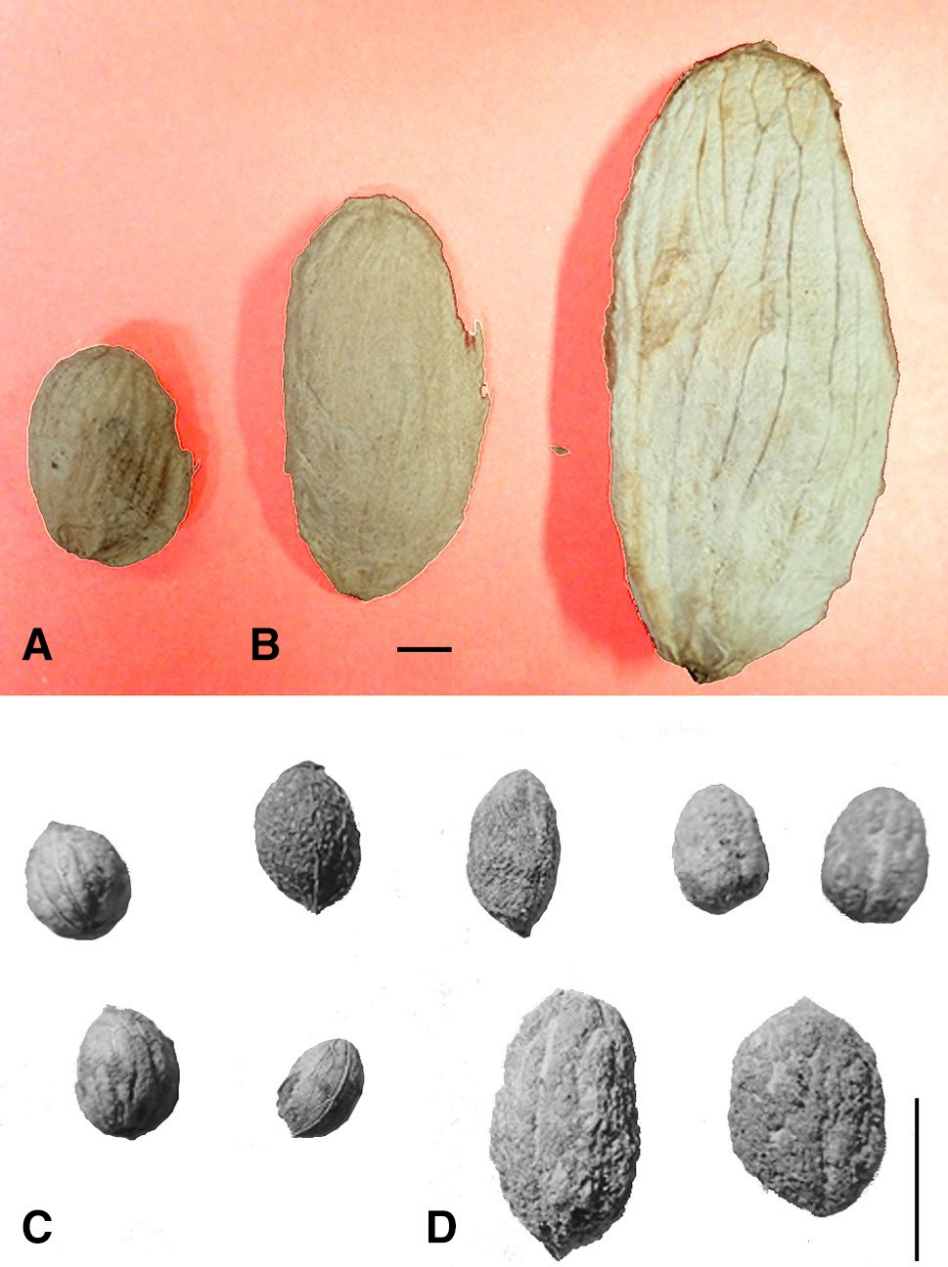
Examples of the contrast between wild and domesticated seeds/endocarps from fruit trees with drupes: **a** wild *Mangifera indica* (collected Maharashtra, India), **b** two domesticated *M. indica*, **c** wild *Olea europaea* ssp. *sylvestris* (collected from Turkey), **d** domesticated *O. europaea* ssp. *europaea*. All specimens in the UCL archaeobotanical reference collection; scale bars 1 cm

Comparisons of modern cultivated and wild growing populations of many fruits establishes that there are differences. Fruits and seeds tend to be larger in domesticated/cultivated fruits by comparison with wild forms. Modern comparisons also allow us to suggest that there tend to be differences in shape as well as size, with domesticated fruit seeds often more elongated (i.e. a higher length: width ratio, hereafter L:W) as well as larger (Fig. 1). This may not be true in all fruits, but it recurs across drupes at least in numerous taxonomically distant taxa, including Sapindales (Anacardiaceae: *Mangifera indica*, Burseraceae: *Canarium indicum*), Laurales (Lauraceae: *Persea americana*), Lamiales (Oleaceae: *Olea europaea*), Rosales (Rosaceae: *Prunus persica*, Rhamnaceae: *Ziziphus jujuba, Z. mauritiana*), as well as berries of Vitales (Vitaceae) and drupe-like berries of the date palm, *Phoenix dactylifera* (Arecaceae). Such differing size and shape tendencies have long been recognized in some taxa, such as Stummer’s (1911) distinction between the short, stubby seeds of wild grapes (*Vitis vinifera* ssp. *sylvestris* and other *Vitis* spp.) and the elongated seeds of domesticates (*V. vinifera* ssp. *vinifera*). While the utility of this simple index for definitively separating early cultivated grapes from wild gathered grapes can be criticized, the fact that wild and domesticated populations fall towards different ends of a morphological spectrum seems clear. Indeed, studies employing geometric morphometrics (GMM) on grapes (*Vitis vinifera*) (Pagnoux et al. 2015; Bacilieri et al. 2017), as well as on other taxa such as olives (*Olea europaea*) (Terral et al. 2004; Newton et al. 2014), plums (*Prunus domestica*) (Ucchesu et al. 2017) and dates (*Phoenix dactylifera*) (Rivera et al. 2014; Gros-Balthazard et al. 2016), which factor out size and focus instead on shape, demonstrate statistically shape differences between wild and cultivated forms of these fruits, as well as among different cultivar groups. Domesticated forms have higher L:W ratios and tend to have more pointed (acute to acuminate) ends. GMM has emerged as a powerful means of separating and classifying different varietal groups of fruits in archaeological material, but it has yet to be applied to estimating rates of evolution under domestication. The present study suggests that there is still some utility in large datasets of simple metrics.

The approach taken in the present study derives from recent assessments of domestication in annual grain crops by plotting time series data of size, identifying episodes of largely directional change and estimating rates of phenotypic evolution in terms of *haldanes* (Purugganan and Fuller 2011; Fuller et al. 2012, 2014). The *haldane* rate [hereafter *h*] represents the shift in the normal statistical distribution of a trait, taking into account not just the mean but the range of the standard deviation (Gingerich 2009). The time unit of the haldane is the generation. The expectation is that the domestication episode represents a period in which change over time in the size/shape parameters of archaeological assemblages is evident, whereas before or after this episode change is absent or non-directional. In the case of the cereal einkorn (*Triticum monococcum*), for example, it is found that on average grain breadth increased 50.4% from 9725 to 6550 bc, whereas since 6550 bc there has been no recognizable change in size (Fuller et al. 2014). This leads to estimated change of 0.02% increase in grain breadth per year or an estimated rate of change of 0.0041 *h* over 3,175 generations of wheat (Fuller et al. 2014). The present study considers a range of fruits and nuts from differing regions of origin, including, China, Japan, West Asia, Oceania and Mesoamerica. The selection of taxa was based on those taxa for which bodies of metrical data appeared to be readily available, with the aim of drawing comparative examples from different regions of origin, and thus highlighting the potential of larger systematic collection of data of this kind. The author gathered as much data as he could find, which often included a wide geographical range. Nevertheless, since geographical dispersal may occur well before the evolutionary process of domestication ends, specimens outside the regions of origin are also relevant. By the inclusion of both taxonomically diverse and geographically varied taxa, patterns and processes that can be considered as convergent evolution and recurrent domestication-related processes of selection can be recognized.

## Materials and methods

Measurements were gathered from the archaeobotanical literature and to a lesser extent through measurements of the author’s own archaeobotanical collections. When measurements were not reported by the original author, but scaled photographs were available, dimensions were estimated from the photographs. Such an approach may inevitably lead to some imprecision and errors of comparability between sources. However the scale of this imprecision is assumed to be much smaller than the standard deviations and extent of difference over time, so the larger, long-term trends of interest here are unlikely to be affected. In addition errors, such inter-observer differences, should be independent of archaeological age and therefore evident chronological trends are unlikely to be result of such errors. In all cases it is essential that we have a mean, standard deviation and sample size and, if possible, maximum and minimum measurements. In cases where the standard deviation is not reported, but sample size, maximum and minimum are available, the standard deviation has been estimated on the basis of sizes of samples following Table 27 of Pearson and Hartley (1976). The taxa, the total quantity of measured specimens and timespan covered by those measurements are summarized in Table 1, while the full dataset is provided in the on-line supplementary (ESM) tables.

**Table 1:** Summary of species considered in the present paper in terms of size change and rates of phenotypic evolution

Species	Total measured specimens	Specimens in inferred domestication episode	Total time span of specimens (period for domestication episode)	Countries of origin of specimens
*Prunus persica* (syn. *Amygdalus persica*)	876	143	5700 bc–ad 575 (3500–1400 bc)	China, Japan, India, Italy, Egypt, UK
*Phoenix dactylifera*	1,410	1,370	5500 bc–ad 1350 (3000 bc–ad 650)	Israel, Oman, Bahrain, Egypt, Mali
*Olea europaea*	984	824	5500 bc–ad 575 (4600–150 bc)	Cyprus, Egypt, Greece, Israel, Jordan
*Castanea crenata*	368	297	7425–700 bc (3000–700 bc)	Japan
*Spondias* sp.	125	125	1500–700 bc	New Guinea
*Canarium* cf. *indicum*	189	189	1500–700 bc	New Guinea
*Persea americana*	264	260	3000 bc–ad 1450 (500 bc–ad 1450)	Mexico

Differentiated are the total sample size versus the sample size included in the inferred domestication episode for which *haldane* rates are calculated. The time ranges of the total dataset versus the domestication episode is also indicated, as is the total geographical spread of samples

**Fig. 2:**
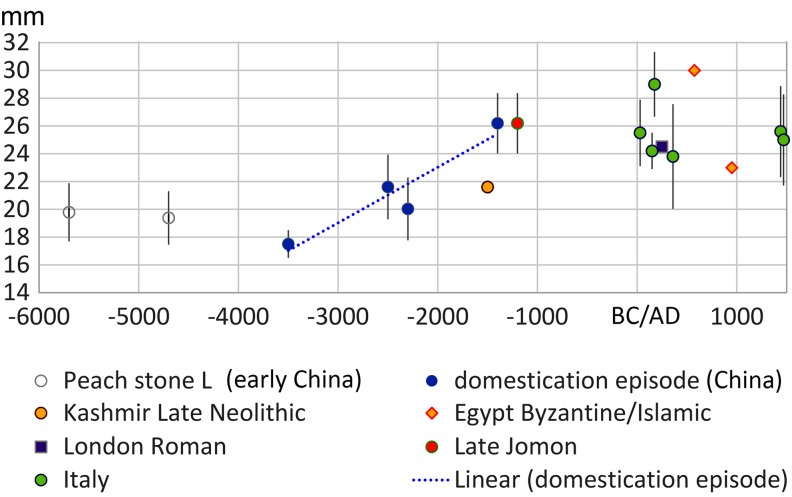
*Prunus persica* stone size over time: average and standard deviations of stone length and plotted by median age estimate. Trend line indicates least squares regression through the inferred domestication episode. Data in ESM Table 1 and Haldane rate estimated in ESM Table 2, ESM Fig. 4

Corrections were made to measurements to make charred and uncharred specimens comparable. As is widely documented, charring is expected to lead to significant shrinkage, whereas size change is less pronounced in waterlogged material or desiccated material. In the cases of *Olea, Phoenix* and *Persea* both charred and uncharred (desiccated) examples were included. In the case of *Prunus persica* both charred and uncharred (waterlogged) material was included. Therefore in these cases a correction factor was applied. Other taxa were all preserved in the same way, either waterlogged (*C. crenata*), or carbonized (*Canarium, Spondias*). Of course, charring will affect seeds variably depending on aspects of charring conditions in the past, which may not be consistent and are unknowable. Muffle furnace charring experiments often suggest reduction in length of ca. 10%, e.g. experiments on *Olea* reported by Terral et al. (2004), or experiments that replicate well-preserved cereal grains (Charles et al. 2015). A 20% reduction can be derived from comparisons of charred and uncharred archaeological data from the same species. For both *Phoenix dactylifera* and *Olea europaea*, I plotted charred and uncharred specimens against time and the calculated the average offset of the linear regressions as −19 and − 20% respectively (Figs. 1, 2 in ESM). I have used a uniform adjustment of − 20%, to make uncharred finds more comparable to charred (for *O. europaea, P. dactylifera, P. americana, P. persica*). This has the advantage of under-estimating size change, whereas a − 10% correction would make temporal trends for size increase even more pronounced.

**Fig. 3:**
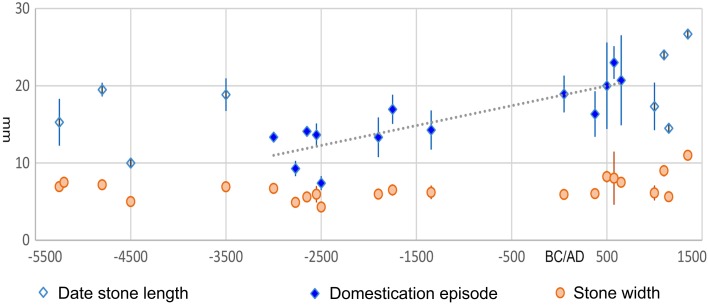
*Phoenix dactylifera* stone size over time: average and standard deviations of stone length and stone width plotted by median age estimate. Trend line indicates least squares regression through the inferred domestication episode. Data in ESM Table 3 and Haldane rate estimated in ESM Table 4, Fig. S5

Measurement summary data were used for a trend analysis in each species. For several larger samples the histogram of individual measurements can be seen to approach a normal distribution (Fig. 3 in ESM), which suggests that it is reasonable to assume most assemblages approach a normal distribution that can be adequately represented by the mean and standard deviation. Means and standard deviations were then plotted against the estimated median age of the sample. This follows the protocols of previous studies (Fuller et al. 2012, 2014; Maeda et al. 2016). When radiocarbon dates for a site or site phase are available the summed probability of calibrated ages was calculated across all radiocarbon dates from the site using the SUM option in OxCal 3.10 (Bronk Ramsey 2005). The median of the range is the single year that has the highest probability of falling within the actual range of occupation, while the one-sigma range of the summed probability is recorded in the ESM data tables. When radiocarbon dates were not available, the age range of site phase was estimated on the basis of the reported cultural phase.

The presence of chronological trends was tested with a Mann–Kendall Trend test, in PAST 3.1 software (Hammer et al. 2001). This tests for directional (monotonic) trends in time series data (Mann 1945). The averages across the entirety of each species dataset was tested, as was the shorter domestication episode. All trends analysed have statistically significant *P* values, which are provided after each ESM data table. This directionality in averages justifies inferring rates of phenotypic evolution from these data.

Domestication episodes, i.e. the period of time over which directional changes in metrics were taking place (Fuller et al. 2014), were identified visually from the plots of the data. Ideally, earlier wild type material shows no significant and directional trends in change, while after the domestication episode metrics are expected to level off. Once a domestication episode period is determined data falling within that period are used to estimate rates of phenotypic change.

Rates of phenotypic change were calculated in terms of the *haldane* rate (*h*), following the method applied previously on several annual grain crops (Fuller et al. 2012, 2014). In the present study we consider change in seed or endocarp as comparable and these plant parts are discussed together by comparing rates of change. This calculation provides the *haldane numerator* which is then divided by the number of generations. In previous domestication studies, focused on cereal and pulses, this was simply the number of years, as such crops are annuals. In the case of long-lived perennial fruit trees, those considered in the present study, generation time is more problematic. For the longer-lived perennials considered here I have assumed a generation time of either 5 or 10 years. Five years is the reported minimal time from seedling to fruit yields in *Phoenix dactylifera* (4 years: Bose and Mitra 1990, p 673) and *Persea americana* (Bose and Mitra 1990, p 557; 4–10 years; Lyle 2006, p 317). Seedling trees of *Olea europaea* may begin producing fruits in 4 to 7 years, reaching full productivity after 8 years (Lyle 2006, p 299), and certainly by 10 years (Murphy 2014). Peaches are reported to bear fruits between the ages of 10 and 20 years in India (Singh 1992, p 179), but potentially as quickly as just 2–3 years (Lyle 2006, p 350), making 5 and 10 year generation estimates both reasonable. *Canarium indicum* is said to produce good crops of nuts at 10 years of age (Howes 1948, p 88). Lyle (2006, p 115) reports *Castanea* seedling trees may produce starting between 4 and 10 years, although Howes (1948, p 137) stated that seedling trees generally take 15–20 years. Similarly a long maturation period, of ca. 15 years is reported for *Mangifera indica* (Bose and Mitra 1990, p 7). Thus generation times of 5 and 10 years can at least be regarded as providing a reasonable bracket for estimating the order of magnitude of the rate of phenotypic change in the taxa considered below, in addition to 15 years for chestnuts.

## Results and discussion

Peaches (*P. persica*) have recently received attention as a probable fruit tree domestication of Neolithic China (Zheng et al. 2014; Weisskopf and Fuller 2014; Stevens et al. 2016). The wild progenitor of the peach is regarded as present from the mountains of central Asia/ far western China through northern China, but it is likely that wild populations have been largely extirpated over much of its former range in the more heavily occupied parts of central and eastern China. The earliest archaeological finds are reported from the Neolithic Yangtze valley back to nearly 6000 bc in the Lower Yangtze valley, with finds in the Yellow river valley by at least the fourth millennium bc. Zheng et al. (2014) reported an increase in fruit stone size amongst later third millennium bc specimens compared to those of the sixth or fifth millennium bc, and a further increase by the later second millennium bc. The large dataset compiled here (Fig. 2), including Roman era and Medieval examples from Europe and Egypt, suggests that these second millennium bc examples from China and Japan were at the end of the endocarp size increase trajectory, but also raises the possibility that early finds in Kashmir represent dispersal out of China before this size change had fully evolved, i.e. the Kashmir specimen falls close to the size of the third millennium bc Chinese examples. Based on an inferred domestication episode between 3500 and 1400 bc, seed length increased at least 35% on average, with a rate of change falling between 0.004 *h* (assuming a 5-year generation) and 0.01215 *h* (assuming a 15-year generation). Subsequent to that episode variation in length is present but shows no directional trends, suggesting that changes were associated with varietal diversification.

Date palms (*P. dactylifera*) originate from a very different ecological and cultural geography, associated in particular with oases in the desert environments of Arabia, the southern Levant and the Sahara. It has long been inferred that the date was brought into cultivation in eastern Arabia around the gulf (e.g. Tengberg 2003, 2012), supported by the recent identification of wild stands in Oman (Gros-Balthazard et al. 2017). Recent genetic studies suggest deep divergence in the genomes of date palms from the central and western Sahara, and north Africa (Hazzouri et al. 2015), raising the possibility of a second centre of domestication, as yet unsupported archaeologically, or a process of introgressive capture (sensu Larson and Fuller 2014) from now extirpated wild palms in the Sahara, perhaps represented by remnant populations on Djerba and Kerkennah islands of Tunisia (Zehdi-Azouzi et al. 2016) and by *P. atlantica* on Cape Verde Islands (Henderson et al. 2006). The Mediterranean sister species, *P. theophrasti* appears to be a true wild species, now extirpated from its former distribution in the Levant, where it was recorded from sixth millennium bc finds in southern Israel (Kislev et al. 2004), the identity of which is confirmed by geometric morphometrics (Rivera et al. 2014; Gros-Balthazard et al. 2016). Middle Holocene finds are restricted to Arabia, Pakistan, Mesopotamia and the Levant, whereas reports from the Nile Valley occur in the early second millennium bc (i.e. the Egyptian Middle Kingdom/Kerma period: Fuller 2015) and more widely after 1500 bc (e.g. Clapham and Stevens 2009), but such date stones were on average smaller or only half the length that domesticated dates were expected to reach by the Iron Age.

A major trend in date stone length increase took place from the third to first millennium bc, although indications of earlier cultivation and possibly domesticated (seed enlarged) assemblages are reported earlier in the Chalcolithic Levant (Fig. 3). This latter trend has been used to calculate a rate of phenotypic change of 0.0014–0.0028 *h*. The date stones from Tuleilat Ghassul in Jordan, dating to ca. 4500 bc, have long been regarded as indicating cultivation as they fall outside the predicted wild range (Zohary and Spiegel-Roy 1975; Weiss 2015), although in terms of size they fit with presumably wild sizes as do those from the Early Bronze Age Qumrum Cave (Liphschitz and Bonani 2001). The date stones from the Cave of the Treasure, ca. 3500 bc median age, however, are significantly larger and suggestive of stones of already domesticated date. These data could indicate more than one domestication process amongst eastern *Phoenix*, or that size increase was delayed in southeastern Arabia due to the wide presence of wild populations in prehistory, with size increase perhaps tied to intensification of date production and extirpation of remnant wild dates from Arabia, over the course of the later Bronze Age.

**Fig. 4:**
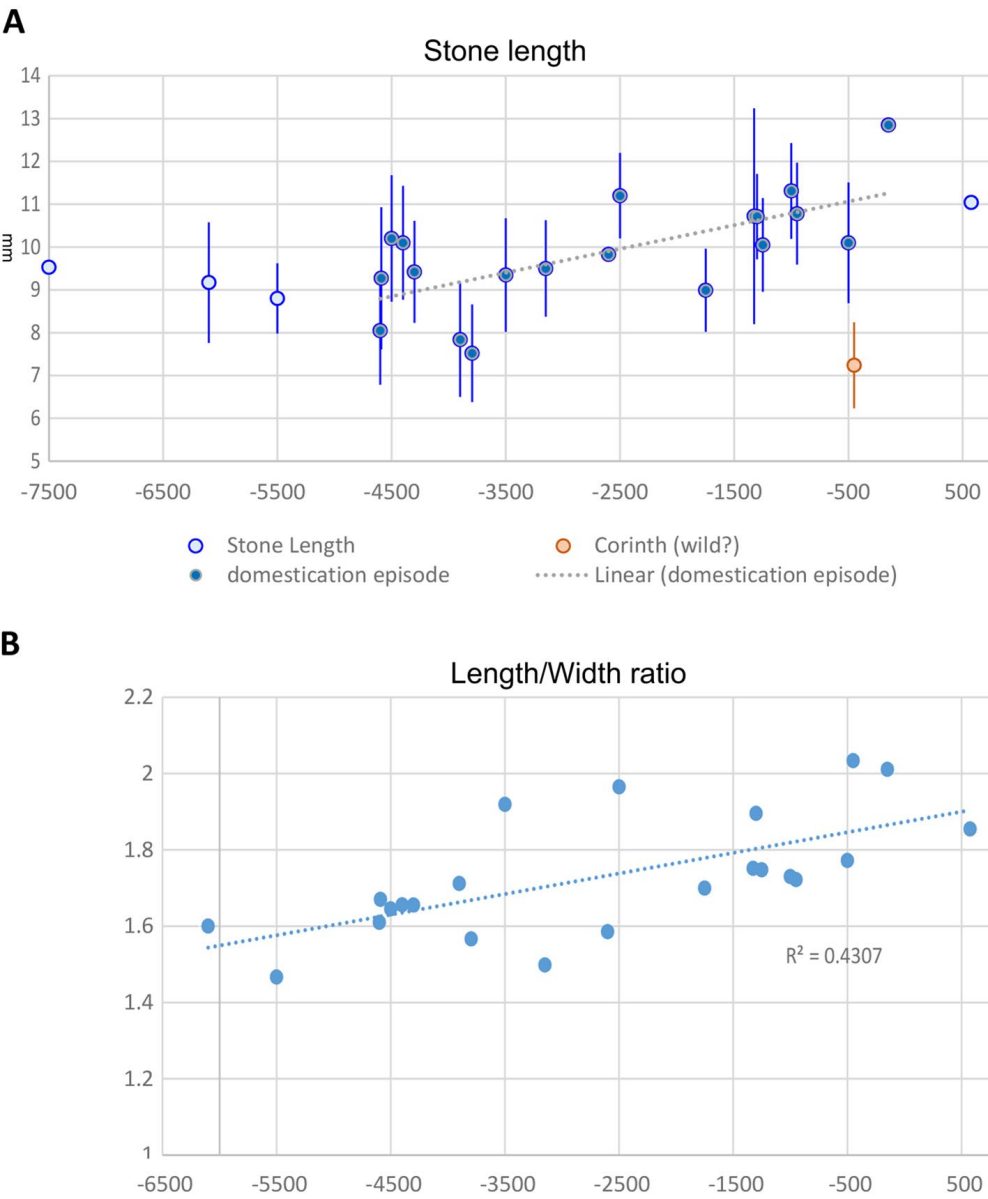
*Olea europaea* stone size over time:** a** average and standard deviations of stone length plotted by median age estimate;** b** length/width ratio over time indicating the trend towards elongation. Data in ESM Table 5 and Haldane rate estimated in ESM Table 6, ESM Fig. 6

Olives (*O. europaea*) are a fruit domesticate of the Mediterranean region, with wild and cultivated populations throughout the basin. Genetic and archaeobotanical studies support the hypothesis of a domestication in the Levant with subsequent dispersal and some introgression from wild populations in the western Mediterranean (Murphy 2014; Newton et al. 2014; Diez et al. 2015; Dighton et al. 2017). Archaeobotanically olives show a gradual increase, starting in the fourth or even fifth millennium bc up to the Iron Age (Fig. 4a), and this is accompanied by a pronounced lengthening in terms of the L/W ratio (Fig. 4b), and an estimated rate of phenotypic change of 0.015–0.003 *h*. Later, small sized olives, like those from Corinth (Bookidis et al. 1999) could represent continued use of some wild populations, although modern cultivars include a few varieties with short stones that overlap with those of wild populations (Terral et al. 2004). The size increase in the eastern Mediterranean documented here is paralleled in Spain by an increase in size and use around the first millennium bc (Buxo i Capdevila 1997; Alonso et al. 2016), which presumably reflects the introduction of domesticated olives from the east.

**Fig. 5:**
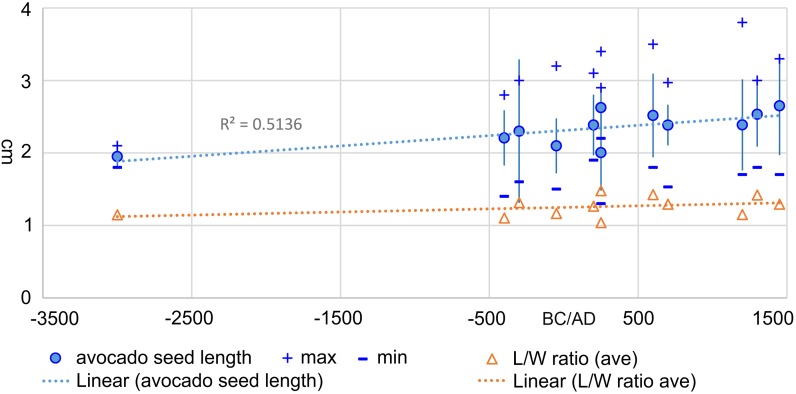
*Persea americana* seed size over time, showing average and standard deviations of length plotted by median age estimate (above), and the average length/width ratio (below). Data from ESM Table 7, with Haldane estimate in ESM Table 8 and ESM Fig. 7

In the New World, a comparable fruit domesticate was the avocado (*Persea americana*), which should demonstrate lengthening of seeds from a nearly spherical ancestral state in wild populations that were native to Southern Mexico or adjacent parts of Mesoamerica (Galindo-Tovar et al. 2008). That these trees were being cultivated before ca. 2500 bc is implied by evidence for their anthropogenic translocation into South America at this date (Pozorski 1979). Avocado endocarps have high archaeological preservation potential, both charred and in desiccated cave contexts in Mexico. However, since the pioneering work of Smith (1966, 1969), there has been little further investigation of the metrics of archaeological avocado. Nevertheless, avocado endocarps provide a basis for considering size change over time. Unfortunately the stratigraphic integrity of these sites has issues, with clear evidence of mixing and intrusiveness in some parts of the stratigraphy. This means it is difficult to be entirely sure about chronology with direct dating. Nevertheless, based on the lower chronology and AMS radiocarbon dates reported by Smith (2005), it is possible to assign most of the measured seed assemblage to probable ages, with the vast majority dating to after 500 bc (Fig. 5). A few reputedly very early specimens (Smith 1966) have been excluded as probable intrusive specimens. Those more safely dated suggest a trend towards increasing seed length between 500 bc and ad 1500, starting sometime after the third millennium bc. The strength of the correlation of size increase with time is rather weak (*r* ^2^ = 0.5136), but supported by the Mann–Kendall trend test, and provides an estimate of 0.0061 to 0.0122 *h*.

**Fig. 6:**
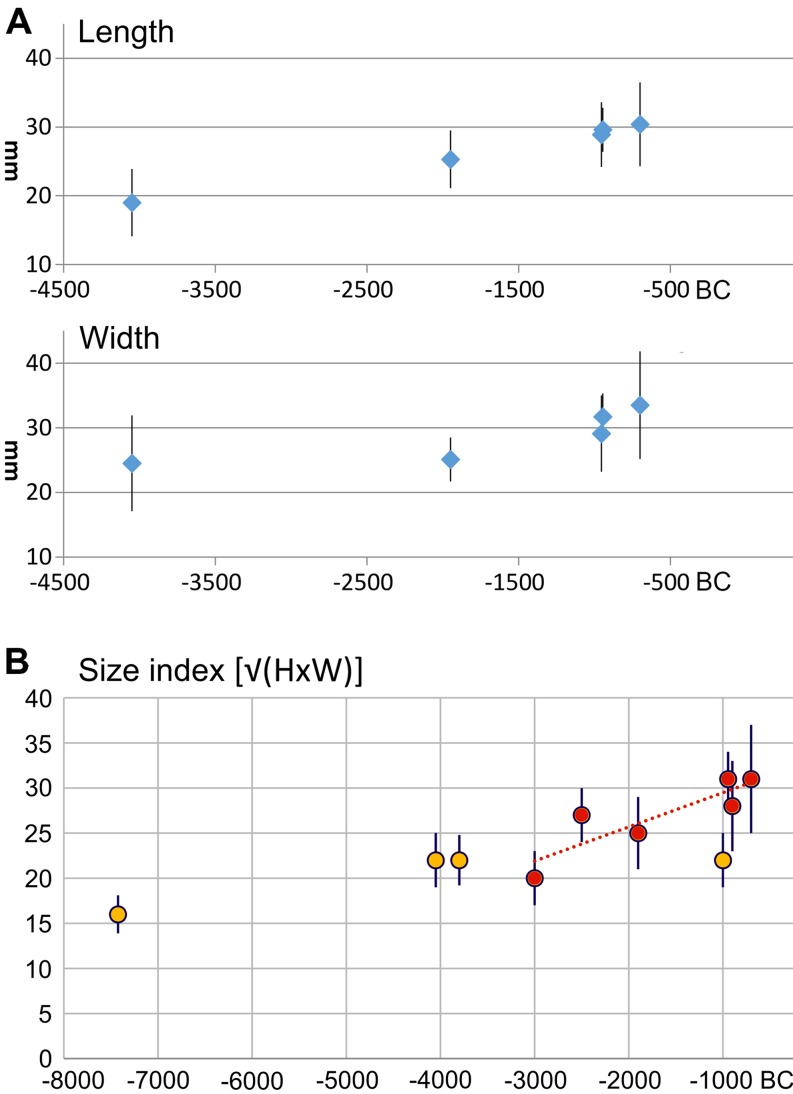
*Castanea crenata* (Japan) nut size over time: **a** average and standard deviations of nut length and width plotted by median age estimate (after Yoshikawa 2011); **b** estimated nut mass (√LxW) from additional sites (after Noshiro and Sasaki 2014). Red circles indicates assemblages in domestication episode; orange circles indicate inferred wild populations. Data in ESM Table 9 and Haldane rates estimated in ESM Table 10, ESM Fig. 8

In Holocene Japan, the Jomon archaeological tradition has increasingly been recognized as a centre for the management of plant resources and even the domestication of at least some of them (e.g. Matsui and Kanehara 2006; Bleed and Matsui 2010; Crawford 2011; Noshiro and Sasaki 2014). Chestnuts trees (*C. crenata*) were important managed resources, and food resources from at least the mid-Holocene, ca. 3000 bc, based on pollen evidence (Noshiro and Sasaki 2014). That these were more than merely managed food resources, but replanted and undergoing selection, i.e. domestication, is indicated by an increase in nut size over course of the Middle to Final Jomon periods, ca. 3000–1000 bc (Yoshikawa 2011; Noshiro and Sasaki 2014). Based on these changes, in both length and a calculated nut mass width (Fig. 6), an evolutionary rate can be estimated as 0.0012 or 0.0019 *h* (with 10-year generation) or as fast as 0.0018 to 0.0029 *h* (15-year generation).

**Fig. 7:**
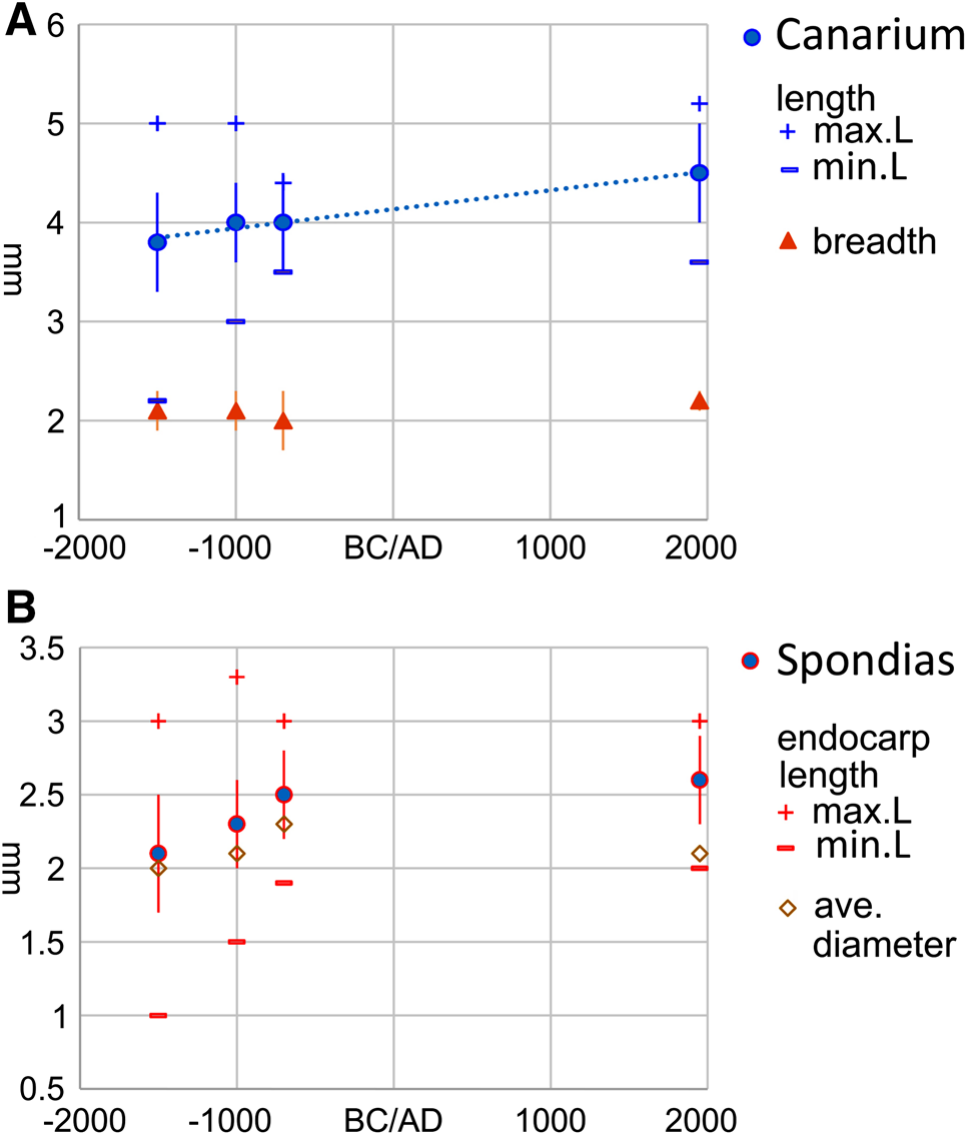
Seed size over time in archaeological fruits from Talepakamalai, Eloua Island, New Guinea: **a** *Canarium* sp. endocarp length (cm); **b** *Spondias* sp. seed length (cm). Data in ESM Tables 11, 12

In Island Southeast Asia and New Guinea fruits and nuts were among the important managed and domesticated forest resources, including notably *Canarium indicum* (e.g. Kennedy 2012), although archaeobotanical data documenting their exploitation and potential domestication processes is limited. Nevertheless data on both *Canarium* sp. endocarps, which contain the edible “nut”, and *Spondias* sp. endocarps from fleshy fruits, have been published from the Lapita period (1500–700 bc) on Eloaua Island, New Guinea, indicating size changes over this period (Lepofsky et al. 1998). Despite the limited sample size in this case a rate of change can still be estimated as 0.0327–0.0654 *h* for *Spondias* and 0.022–0044 *h* for *Canarium* (Fig. 7).

### Comparing rates of domestication and implications for selection

**Fig. 8:**
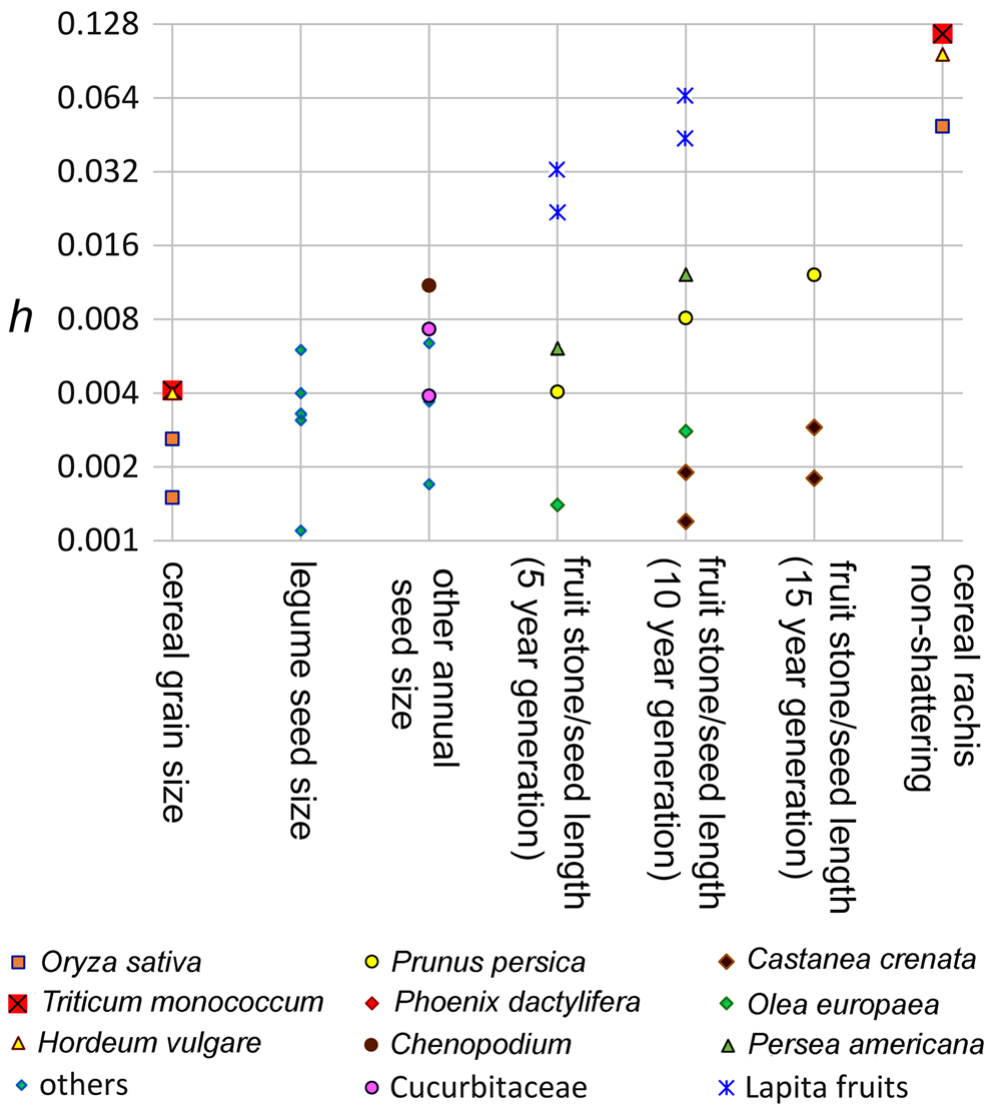
Comparison of estimates Haldane rates of phenotypic evolution during domestication. Rates of change in fruit crops from the present paper are shown based on various generation time assumptions. These are plotted for comparison with Haldane estimates for seed size and non-shattering in annual crops (from Fuller et al. 2014). Pulses include *Vigna radiata, Lens culinaris, Pisum sativum, Cicer arietinum* and *Glycine max;* Cucurbitaceae include *Cucurbita pepo* and *Cucumis melo;* other annuals include *Helianthus annus, Iva annua*

It is notable that the calculated rates for these various perennial fruits is comparable in order of magnitude to rates of seed size increase recorded in annual seed crops. Size change in several cereals, pulses and additional seed crops was compiled in recent studies (Fuller et al. 2012, 2014), and provided estimates of Haldane rates for phenotypic evolution during domestication. Rates are compared across the fruits of the present paper and published annual crop traits (Fig. 8). These data indicate that while fruit size increase was of a comparable order of magnitude, in many cases our rates of change are higher than those recorded in annual crops. This is at first counter intuitive as perennial taxa like trees are often regarded as harder to domesticate. However, this can perhaps be understood as partly due a key difference in selection mechanism. For the most part it appears likely that grain size increase in annual seed crops evolved gradually through a process of unconscious selection (Fuller 2007; Fuller et al. 2014). In addition the level of selection was kept somewhat low by the problem that the cost of selection is additive across all traits under selection and very strong selection increases the risk of population extinction (Allaby et al. 2016). By contrast with staples like cereals, stronger selection pressures that risk local crop failure would be more tolerable in non-staple fruits. In addition, the mechanism for selecting for larger fruits plausibly had a conscious element, i.e. favouring seeds from larger fruits for planting while smaller fruited trees were preferentially removed and pruned. That this planting must have involved some planting of seeds, and not just vegetative propagation, is implied by the protracted periods of gradual size change, suggesting gradual accumulation of mutations for larger fruits, as already inferred by Weiss (2015).

As the selection processes differed between cereal grains and fruit stones, so too did the likely underlying genetic mechanisms involved. In cereals it is clear that selection favoured grain girth, width and thickness (Fuller et al. 2014). In the tree fruits by contrast it was seed/endocarp length. Increase in seed/endocarp length, without comparable increase in width, meant that the surface area of the seed/endocarp increased while the overall of content of the seed, in terms of volume of nutrients did not, i.e. there was an increase in surface area: volume ratio. This implies that there could be an increase in size of the fruit without the need for comparable increased physiological investment in the seed contents. While the increase in seed content was important for the competitiveness of seedlings of arable annuals, we can assume the cultivated perennial fruit would have been more carefully tended as seedlings, as their cultivation represented a long term investment, on the scale of at least a decade before returns from fruit production could be expected. In fruits the increase in seed length with less increase in volume would allow additional metabolites to be invested in fruit flesh, both increasing its amount and potentially increasing anthropogenically favoured nutrients.

**Fig. 9:**
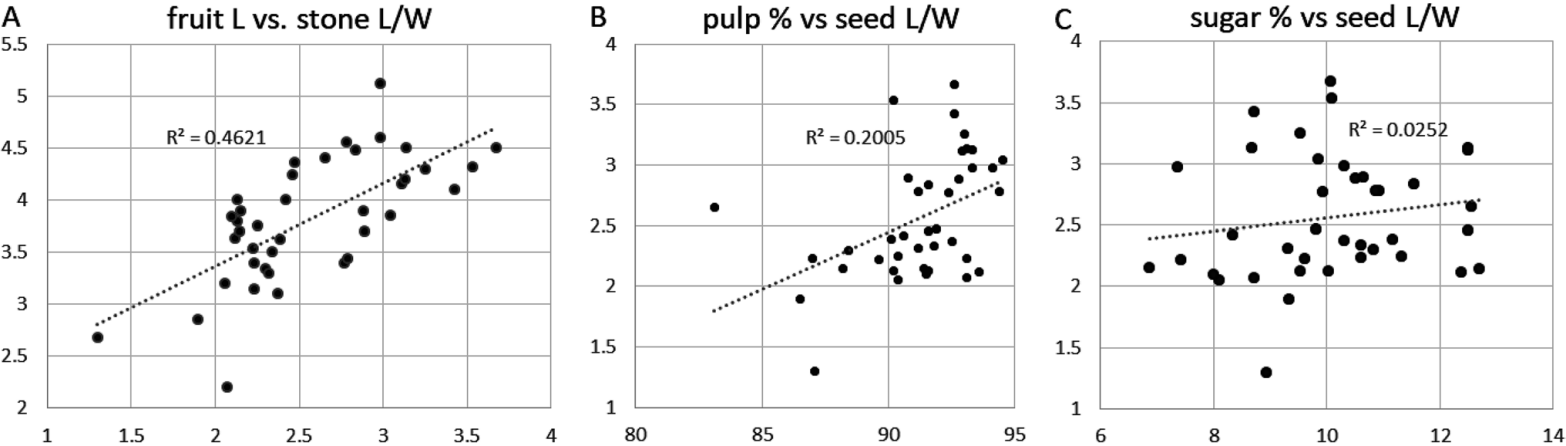
Correlations between fruit size, edible contents and fruit stone illustrated by data from modern Indian jujube (*Ziziphus mauritiana*); each data point represents the average from a different variety (data from Bal and Uppal 1992). **a** Scatter-plot of average fruit length versus average stone length/width (L/W) ratio. **b** Scatter-plot percentage of average fruit pulp versus average endocarp L/W ratio. **c** Scatter-plot percentage sugar content versus average endocarp L/W ratio

If the latter hypothesis is correct then we would expect a positive correlation of elongated fruits, i.e. higher L/W ratio with increased fruit content, i.e. amount of pulp or properties such as sugar content. Data to test this were difficult to find, but in the case of Indian jujube (“ber”) a large quantitative dataset on varietal variation (Bal and Uppal 1992) offered an opportunity to explore such correlations. In this dataset average values are reported for 32 traditional varieties of *Ziziphus mauritiana* tree cultivars in India. Among these varietal averages it is clear that there is a correlation between overall fruit length and the L:W ratio of the stone (Fig. 9a), i.e. longer fruits had more elongate stones (endocarps), as expected from observations of reference material (Fig. 1) and from the archaeobotanical evidence of peaches, dates and olives. More intriguingly there appears to be a positive correlation between elongated stone L/W ratio and the percentage of fruit pulp, with more pulp on more elongated fruit (Fig. 9b). There may also be a very weak correlation between stone length and sugar content (Fig. 9c), although one might expect there to be much variation in how sweet fruits are due to various cultural selection patterns based on other aspects of taste. Nevertheless, the hypothesis is, for fleshy drupes at least, that human selection for increasing edible fruit flesh selected morphologically for elongation of seed/stone length. Thus conscious selection on the obvious trait of fruit food value, and perhaps sweetness, could have driven the unintended evolution in seed/stone size and shape.

## Concluding remarks

The comparative data presented in this paper highlight that morphological evolution of tree fruits can be documented through archaeobotanical evidence using simple metrics and when large time series are available. The sample sizes for most of these fruits is still modest and this calls for the recording and publication of seed size data from archaeobotanical fruits and nuts. Larger systematic measurement programs can test and refine the apparent trends identified here, and overcome likely problems of precision in the available data gathered here. Nevertheless, the data analysed here indicate significant chronological trends and suggest parallel patterns of evolution across several fruit/nut species. These data indicate similar rates of change in fruits and nuts drawn from taxonomically and geographically diverse examples. In addition we see similar kinds of shape change as seeds or stones of fleshy fruits get longer and more pointed (more attenuated). This can be regarded as part of a domestication syndrome in fleshy fruits, driven perhaps by selection for more fruit flesh that had the unintended effect of making seeds/endocarps longer and more acute. That this gradual evolution took place implies that reproduction by seed, with attendant mutation and recombination, played an important role in early fruit tree reproduction, the “intermediate” grade of fruit cultivation explored by Goldschmidt (2013; see also Weiss 2015). In this case we can infer that routine vegetative propagation is likely to have only begun around or after the end of the domestication episode, i.e. ca. 1000 bc for peaches and date palms, ca. 2000 bc for olives. It is at this stage that the diversification and maintenance of distinct varieties of fruit can be suggested to have become dominant over primary domestication processes. Cultivation of perennial fruits was an important way in which agricultural societies tamed the landscape, and archaeobotanical evidence has much to contribute to documenting this by juxtaposing the evolution of cultural trees and systems of land use. The patterns identified in this pilot study argue for more large scale and systematic collection of archaeological fruit metrics.

## Electronic supplementary material

Below is the link to the electronic supplementary material.

Supplementary material 1 (DOCX 170 KB)
