# Supplementary material for: Long and attenuated: comparative trends in the domestication of tree fruits
Source: Veg Hist Archaeobot. 2017 Dec 9;27(1):165–76. doi: 10.1007/s00334-017-0659-2 (PMC6954012; doi:10.1007/s00334-017-0659-2)
Supplement: Supplementary file 1 — Supplementary material 1 (DOCX 170 KB) [file 334_2017_659_MOESM1_ESM.docx]

**Online Supplementary Material for Fuller “Long and attenuated: comparative trends in the domestication of tree fruits”**

**
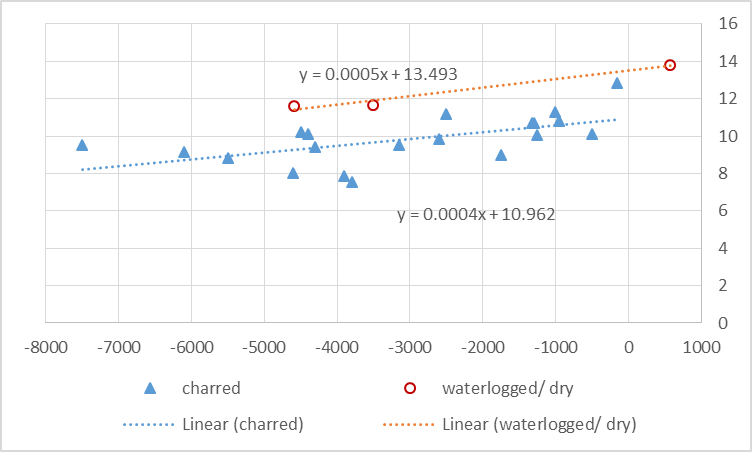
**

**Fig. 1** Plot of charred and desiccated *Olea* stones against time. The difference between the trend line formulae suggest that charred specimens approach ca. 81% of the length of uncharred ones (i.e. 10.962/13.493). For data, see Table S5

**
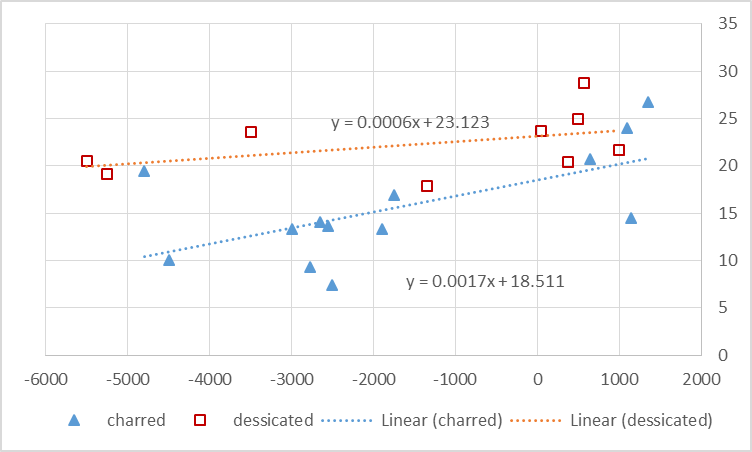
**

**Fig. 2** Plot of charred and desiccated *Phoenix* stones against time. The difference between the trend line formulae suggest that charred specimens approach ca. 80% the length the length of uncharred ones (i.e. 18.511/23.123)

A
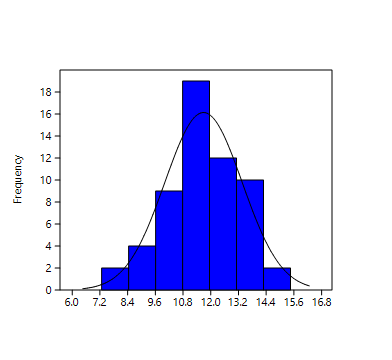
 B
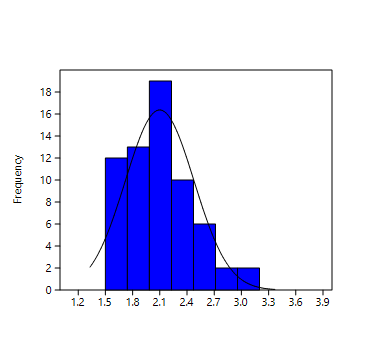


C
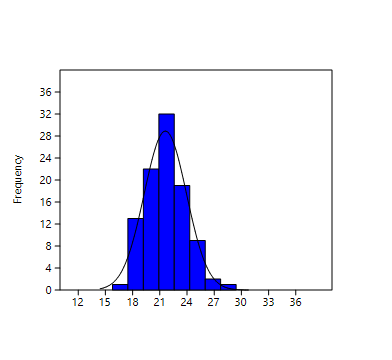
 D
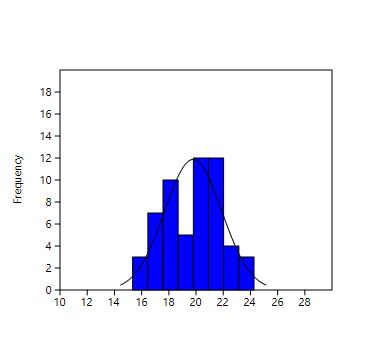


**Fig. 3** Examples of assemblages that demonstrate approximation of normal distribution. A. *Olea europaea* length (mm) from Cave of the Treasure (7 bins); B. *Persea* stone length (cm) from Coxcatlan Cave TC50 VI (in 7 bins); C. *Prunus persica* stone length from Maoshan (in 8 bins); D. *Prunus persica* stone length (mm) from Kuahuqiao (in 8 bins)

**Table 1** *Prunus persica* metrical data

| **Country** | **Ssite** | **Time** | **N** | **L.mean** | **L.min** | **L.max** | **Stdev** | **W.ave** | **W.min** | **W.max** | **W.std** | **Source** |
| --- | --- | --- | --- | --- | --- | --- | --- | --- | --- | --- | --- | --- |
| China: Zhejiang | Kuahuqaio | *-5700* | 56 | 19.78768 | 15.34 | 24.26 | 2.097033 | 15.52018 | 13.07 | 18 | 1.131121 | Zheng et al. 2014 |
| China: Zhejiang | Tianluoshan | -4700 | 9 | 19.38 | 16.52 | 23.28 | 1.923421 | 15.25333 | 13.11 | 17.44 | 1.246224 | Zheng et al. 2014 |
| China: Jiangsu | Dongshan-cun | -3500 | 13 | 17.5 | 15 | 20 | 1 | 11.8 | 10 | 14.8 | 1.849324 | Qin L, Deng Z, Fuller DQ (n.d.) |
| China: Zhejiang | Maosahan | -2500 | 99 | 21.61061 | 15.78 | 29.42 | 2.329027 | 16.85889 | 13.27 | 21.27 | 1.553275 | Zheng et al. 2014 |
| China: Zhejiang | Bianjiashan | -2300 | 23 | 20.02913 | 17.14 | 25.49 | 2.265468 | 15.6013 | 13.26 | 18.02 | 1.414147 | Zheng et al. 2014 |
| China: Zhejiang | Qinshan-yang | -1400 | 7 | 26.19286 | 24.08 | 29.96 | 2.174164 | 18.78714 | 17.38 | 21.82 | 1.516187 | Zheng et al. 2014 |
| India: Kashmir | Burzahom | -1500 | 1 | 21.6 |  |  | 0 |  |  |  |  | Lone et al. 1993 |
| Japan | Ikiriki | -1200 | 8 | 26.19286 | 18.9 | 25.1 | 2.174164 | 17.8375 | 16.3 | 20.2 | 1.358505 | Zheng et al. 2014 |
| Italy | Modena | 30 | 106 | 25.5 | 18 | 30.1 | 2.392865 | 19.6 | 12.6 | 23 | 2.056677 | Sadori et al. 2009 |
| Italy | Aquileia | 150 | 162 | 24.2 | 21 | 28 | 1.308201 | 18.1 | 16 | 21 | 0.934429 | Sadori et al. 2009 |
| Italy | Vercelli | 175 | 18 | 29 | 25.5 | 34 | 2.335126 | 21.9 | 18.5 | 26 | 2.060406 | Sadori et al. 2009 |
| Italy | Rome | 360 | 253 | 23.8 | 14 | 35.3 | 3.772516 | 19 | 11.7 | 27.8 | 2.851526 | Sadori et al. 2009 |
| Italy | Imola | 1440 | 92 | 25.6 | 15.1 | 31.3 | 3.269233 | 20.3 | 13 | 25.7 | 2.562918 | Sadori et al. 2009 |
| Italy | Moncalieri | 1470 | 26 | 25 | 18 | 31 | 3.279251 | 19.8 | 17 | 22.5 | 1.387375 | Sadori et al. 2009 |
| England | London | 250 | 1 | 24.5 |  |  | 0 | 20.5 |  |  |  | Willcox 1977 |
| Egypt | Quseir al-Qadim | 950 | 1 | 23 |  |  |  | 16 |  |  |  | Van der Veen 2011 |
| Egypt | Kom el-Nana | 575 | 1 | 30 |  |  | 0 | 22.35 |  |  |  | Smith 2003 |

Mann-Kendall trend test of ave.L. all *Prunus persica, p*(no trend): 0.020958*

Mann-Kendall trend test of ave.L. *Prunus persica,* 3500-1200 BC*, p*(no trend): 0.028*

**Table 2** *Prunus persica* domestication episode data for haldanes calculation

| **Site** | **Age** | **n** | **L.ave** | **Stdev** | **s-terms (stdev^2^x (n-1))** | **n-terms (n-1)** | **Generations (10 yr)** | **x/σ** |
| --- | --- | --- | --- | --- | --- | --- | --- | --- |
| Dongshancun | -3500 | 13 | 17.5 | 1 | 12 | 12 | 0 | 3.960318 |
| Maosahan | -2500 | 99 | 21.61061 | 2.329027 | 531.588 | 98 | 120 | 3.576138 |
| Bianjiashan | -2300 | 23 | 20.02913 | 2.265468 | 112.9116 | 22 | 220 | 4.416144 |
| Qinshanyang | -1400 | 7 | 26.19286 | 2.174164 | 28.36194 | 6 | 240 | 4.092968 |
|  |  |  |  | **Sum:** | 684.8615 | 138 |  |  |
|  |  |  |  | σ (s sum/ n sum) | 4.962764 |  |  |  |

**Fig. 4** Graph of Haldane rate estimate for *Prunus persica*

**Table 3** *Phoenix dactylifera* metrical data

| **Region** | **Site** | **Preserv./ correction** | **Age median** | **n** | **L ave** | **L min** | **L max** | **Stdev** | **n** | **B ave** | **B min** | **B max** | **Stdev** | **Source** |
| --- | --- | --- | --- | --- | --- | --- | --- | --- | --- | --- | --- | --- | --- | --- |
| Pakistan | Mehrgarh IB | Mineral.?): -20% | -5500 | 2 | 16.44 | 16.32 | 16.56 | 0.1697 | 2 | 6.08 | 6 | 6.16 | 0.1131 | Costantini 1985; Clapham and Stevens 2009 |
| Jordan | Tuleilat Ghassul | charred | -4500 | 7 | 10 | 9 | 11 | 0.63 |  | 5 | 5 | 5 | 0 | Lipshitz & Bonani 2001 |
| Kuwait | Sabiyah H3 | mineralized: -20% | -5250 | 3 | 15.28 | 12 | 18 | 3.0353 | 3 | 6.933333 | 6.16 | 7.6 | 0.7259 | Beech 2003; Clapham and Stevens 2009 |
| Iran | Tepe Gaz Tavila | charred | -5200 |  |  |  |  |  | 1 | 7.5 |  |  | 0 | Beech 2003; Clapham and Stevens 2009 |
| UAE | Dalma | charred/impressions | -4800 | 2 | 19.5 | 19 | 20 | 0.8862 | 4 | 7.175 | 7 | 7.6 | 0.2914 | Beech 2003; Clapham and Stevens 2009 |
| Israel | Cave of the Treasure | desiccated: -20% | -3500 | 9 | 18.84444 | 15.92 | 21.52 | 2.1106 | 9 | 6.924444 | 6.48 | 7.68 | 0.3625 | Zaitschek 1980 (estimated from image) |
| UAE | Hili 8 IB | charred | -3000 | 5 | *13.35* | 12.7 | 14 | *0.5589* | 5 | *6.7* | 6.2 | 7.2 | *0.4299* | Costantini 1985; Clapham and Stevens 2009 |
| Israel | Qumran Cave | charred | -2770 | 7 | 9.28 | 8 | 11 | 0.994 |  | 4.9 | 4.5 | 6 | 0.494 | Lipshitz & Bonani 2001 |
| Iraq | Ur | charred | -2650 | 1 | 14.1 |  |  |  | 4 | 5.6 | 4.8 | 7.2 | 1.0677 | Costantini 1985; Clapham and Stevens 2009 |
| UAE | Hili 8 IIC | charred | -2550 | 15 | *13.65* | 11.1 | 16.2 | *1.4690* | 15 | *5.95* | 5.1 | 6.8 | *0.4897* | Costantini 1985; Clapham and Stevens 2009 |
| Pakistan | Miri Qalat | charred | -2500 | 16 | 7.4 | 5.5 | 8.7 | 0.9060 | 16 | 4.3 | 3.2 | 5.2 | 0.5663 | Beech 2003; Clapham and Stevens 2009 |
| Oman | R’as al-Jinz 2 | charred | -2300 | 39 | 14.68 | 9.21 | 18.15 |  |  |  |  |  |  | Costantini and Audisio 2000 |
| Bahrain | Saar | charred | -1900 | 16 | 13.33 | 10.4 | 19.5 | 2.5765 | 16 | 5.97 | 4.6 | 7.2 | 0.7361 | Nesbitt 1993; Clapham and Stevens 2009 |
| Kuwait | Failaka | charred | -1750 | 4 | 16.95 | 14.8 | 18.7 | 1.8944 | 4 | 6.51 | 5.7 | 7.5 | 0.8743 | Rowley-Conwy 1987; Clapham and Stevens 2009 |
| Egypt | Amarna | desiccated: -20% | -1345 | 945 | 14.272 | 7.704 | 24.024 | 2.5289 | 945 | 6.2 | 3.592 | 7.904 | 0.6682 | Clapham and Stevens 2009 |
| Israel | Qumran Cave | desiccated: -20% | 50 | 6 | 18.928 | 18.4 | 23.2 | 2.384 |  | 5.928 | 5.6 | 6.4 | 0.2744 | Lipshitz & Bonani 2001 |
| Egypt | Qasr Ibrim | desiccated: -20% | 375 | 337 | 16.34 | 9.20 | 26.40 | 2.95 | 337 | 6.03 | 3.6 | 8.00 | 0.75 | Clapham and Stevens 2009 |
| Israel | En Boqeq Castle | charred | 500 | 12 | 20.00 | 12.00 | 25.60 | 5.59 |  | 8.24 | 5.6 | 16.00 | 3.44 | Lipshitz & Bonani 2001 |
| Egypt | Kom el-Nana | desiccated: -20% | 575 | 2 | 23.00 | 21.50 | 24.50 | 2.12 | 2 | 8.04 | 8 | 8.08 | 0.06 | Smith 2003 |
| Israel | Evrona | charred | 650 | 4 | 20.70 | 15.00 | 27.00 | 5.83 | 4 | 7.50 | 6 | 8 | 0.97 | Lipshitz & Bonani 2001 |
| Egypt | Quesir al-Qadim | desiccated: -20% | 1000 | 13 | 17.32 | 13.60 | 23.20 | 3.08 | 13 | 6.12 | 5.6 | 7.2 | 0.5 | van der Veen 2011 |
| Syria | Qaryat Medad | Charred | 1150 | 1 | 14.5 |  |  |  | 4 | 5.625 | 5 | 6.5 | 0.75 | Samuel 2001 |
| Mali | Gao | Charred | 1100 | 1 | 24 |  |  |  | 1 | 9 |  |  |  | Fuller 2000 |
| Mali | Essouk | Charred | 1350 | 1 | 26.7 |  |  |  | 1 | 11 |  |  |  | Nixon et al 2011 |

Mann-Kendall trend test of ave.L. all *Phoenix dactylifera, p*(no trend): 0.0094809*

Mann-Kendall trend test of ave.L. *Phoenix dactylifera,* 3000 BC-AD 650 *, p*(no trend): 0.0018617*

**Table 4** *Phoenix dactylifera* domestication episode data for haldanes calculation

| **Site** | **Age** | **n** | **L.ave** | **stdev** | **s-terms (stdev^2^x (n-1))** | **n-terms (n-1)** | **generations  (5 yr)** | **x/σ** |
| --- | --- | --- | --- | --- | --- | --- | --- | --- |
| Hili 8 IB | -3000 | 5 | *13.35* | *0.55892* | 4 | 1.249549 | 0 | 1.886788 |
| Qumran Cave | -2770 | 7 | 9.28 | 0.994 | 6 | 5.928216 | 46 | 1.311565 |
| Ur | -2650 | 1 | 14.1 |  | 0 | 0 | 70 | 1.992788 |
| Hili 8 IIC | -2550 | 15 | *13.65* | *1.46897* | 14 | 30.21005 | 90 | 1.929188 |
| Miri Qalat | -2500 | 16 | 7.4 | 0.906007 | 15 | 12.31274 | 100 | 1.04586 |
| R’as al-Jinz 2 | -2300 | 39 | 14.68 |  |  |  |  |  |
| Saar | -1900 | 16 | 13.33 | 2.576459 | 15 | 99.57208 | 220 | 1.883962 |
| Failaka | -1750 | 4 | 16.95 | 1.894353 | 3 | 10.76572 | 250 | 2.395585 |
| Amarna | -1345 | 945 | 14.272 | 2.528919 | 944 | 6037.288 | 331 | 2.017097 |
| Qumran Cave | 50 | 6 | 18.928 | 2.384 | 5 | 28.41728 | 610 | 2.675141 |
| Qasr Ibrim | 375 | 337 | 16.336 | 2.950652 | 336 | 2925.333 | 675 | 2.308807 |
| En Boqeq Castle | 500 | 12 | 20 | 5.592 | 11 | 343.9751 | 700 | 2.826649 |
| Kom el-Nana | 575 | 2 | 23 | 2.12132 | 1 | 4.5 | 715 | 3.250646 |
| Evrona | 650 | 4 | 20.7 | 5.82878 | 3 | 101.924 | 730 | 2.925582 |
|  |  |  |  | **Sum:** | 1357 | 9601.475 |  |  |
|  |  |  |  | σ (s sum/ n sum) | 7.075516 |  |  |  |

Mann-Kendall trend test of ave.L. all *Phoenix dactylifera, p*(no trend): 0.0094809*

Mann-Kendall trend test of ave.L. *Phoenix dactylifera,* 3000 BC-AD 650 *, p*(no trend): 0.0018617*

**Fig. 5** Graph of Haldane rate estimate for *Phoenix dactylifera* (5 year generation)

**Table 5** *Olea europaea* metrical data

| Country | Site | Phase | Age | Preser-vation | N | L.ave | L.min | L.max | stdev | Th. Ave | Th. Min | Th. max | stdev | L/Th | Source |
| --- | --- | --- | --- | --- | --- | --- | --- | --- | --- | --- | --- | --- | --- | --- | --- |
| Cyprus | Khirokitia | Neolithic | -5500 | Charred | 1 | 8.8 |  |  | 0 | 6 |  |  |  | 1.466667 | Miller 1984 |
| Israel | Kfar Samir | Chalcolithic | -4590 | -20% (waterlog.) | 100 | 9.272 | 6 | 13.064 | 1.408521 | 5.552 | 4.264 | 7.2 | 0.585421 | 1.670029 | Liphschitz et al 2013 |
| Israel | Nahal Zehora II | Chalcolithic | -4600 | charred | 10 | 8.05 | 7 | 9.5 | 0.82 | 5 | 3.5 | 6 | 0.97 | 1.61 | Liphschitz and Bonani 2000 |
| Jordan | Teleilat Ghassul | Chalcolithic | -4500 | Charred | 10 | 10.2 | *7.1* | 13.3 | 1.66 | 6.2 | 4.3 | 6.5 | 0.63 | 1.645161 | Liphschitz et al 2013 |
| Jordan | Abu Hamid | Chalcolithic | -4400 | Charred | 4 | 10.1 | 8.2 | 10.8 | *1.2629* | 6.1 | 5.3 | 6.7 | *0.68002* | 1.655738 | Liphschitz et al 2013 |
| Jordan | Pella | Chalcolithic | -4300 | charred | 34 | 9.42 | *6.5* | *12* | 1.476 | 5.69 | *3.9* | *7.5* | 0.755 | 1.655536 | Dighton et al 2017 |
| Israel | Nevallat | Chalcolithic | -3900 | Charred | 36 | 7.84 | 5.5 | 11.5 | 1.33 | 4.58 | 3.5 | 6.5 | 0.69 |  | Van den Brink et al 2001 |
| Israel | Shoham | Chalcolithic | -3795 | Charred | 23 | 7.52 | 5 | 10 | 1.19 | 4.8 | 3.5 | 6 | 0.7 | 1.566667 | Liphschitz et al 2013 |
| Israel | Cave of the Treasure | Chalcolithic | -3500 | -20% (desicc.) | 58 | 9.35 | 5.82 | 12.36 | 1.34 | 4.87 | 3.64 | 5.82 | 0.50 | 1.918919 | Zaitchek 1980 [from photo] |
| Jordan | Pella | EBA | -3150 | charred | 5 | 9.50 | *7.20* | *10.50* | 1.14 | 6.34 | *5.90* | *6.80* | 0.61 | 1.498423 | Dighton et al 2017 |
| Jordan | Tell esh Shuna | EBA | -2600 | Charred | 70 | 9.83 | 7 | 13.3 | 1.324999 | 6.2 | 5.3 | 7.2 | 0.399603 | 1.585253 | Liphschitz et al 2013 |
| Israel | Lachish | EBA | -2500 | Charred | 20 | 11.2 | 9 | 13.2 | 1.124513 | 5.7 | 5 | 6.2 | 0.321289 | 1.964912 | Helbaek 1958 |
| Jordan | Pella | MBA | -1750 | charred | 1 | 8.99 |  |  |  | 5.29 |  |  |  | 1.699433 | Dighton et al 2017 |
| Jordan | Pella | LBA | -1325 | charred | 41 | 10.72 | *8.4* | *13* | 0.999 | 6.12 | *5* | *7.25* | 0.529 | 1.751634 | Dighton et al 2017 |
| Israel | Tel Jerisheh | LBA | -1300 | Charred | 28 | 10.71 | 9 | 12.5 | 0.97 | 5.65 | 5 | 6.5 | 0.45 | 1.895575 | Liphschitz et al 2013 |
| Greece | Tiryns | LBA | -1250 | Charred | 25 | *10.05* | 5.1 | 15 | 2.51868 | *5.75* | 3.6 | 7.9 | 1.093972 | 1.747826 | Runnels & Hansen 1986 |
| Israel | Tell Keisan | Iron Age | -1000 | Charred | 100 | 11.31 | 8.33 | 13.33 | 0.996971 | 6.46 | 4.83 | 7.17 | 0.466583 | 1.73 | Kislev 1995 |
| Jordan | Pella | Iron Age | -950 | charred | 171 | 10.78 | *7.4* | *13* | 1.095 | 6.26 | *4.3* | *7.8* | 0.529 | 1.722045 | Dighton et al 2017 |
| Israel | Lachish | Iron Age | -500 | Charred | 25 | 10.1 | 8.2 | 12.6 | 1.119413 | 5.7 | 5.1 | 6.4 | 0.330736 | 1.77193 | Helbaek 1958 |
| Israel | Gamla | Hellenistic | -150 | charred | 64 | 12.85 | 10 | 14.5 | 1.19 | 6.39 | 5 | 8 | 0.7 | 2.010955 | Liphschitz et al 2013 |
| Egypt | Kom el-Nana | Byzantine | 575 | -20% (desicc.) | 10 | 11.04 | 8.96 | 13.76 | 1.40905 | 5.952 | 4.8 | 7.36 | 0.694563 | 1.854839 | Smith 2003, Fig. 3.21 |
| Greece | Corinth | Iron Age | -450 | charred | 5 | 7.24 | 5.6 | 8 | 1.004 | 3.56 | 2.6 | 4.2 | 0.6 | 2.033708 | Bookidis et al 1999: Fig. 22 |

Mann-Kendall trend test of ave.L. all *Olea europaea, p*(no trend): 0.014397*

Mann-Kendall trend test of ave.L. Olea inferred domestication episode, p(no trend): 0.017221*

**Table 6** *Olea europea* domestication episode data for haldanes calculation

|  | **Site** | **Period** | **Median Age** | **n** | **L. ave** | **L. stdev** | **s-terms (stdev^2^x (n-1))** | **n-terms (n-1)** | **Gener-ations (10-yr)** | **x/σ** | **Site** | **Period** |
| --- | --- | --- | --- | --- | --- | --- | --- | --- | --- | --- | --- | --- |
| Israel | Nahal Zehora II | Chalcolithic | -4600 | 10 | 8.05 | 7 | 9.5 | 0.82 | 6.0516 | 9 | 0 | 5.034316 |
| Israel | Kfar Samir | Chalcolithic | -4590 | 100 | 9.272 | 6 | 13.064 | 1.408521 | 196.4092 | 99 | 10 | 5.798531 |
| Jordan | Teleilat Ghassul | Chalcolithic | -4500 | 10 | 10.2 | *7.1* | 13.3 | 1.66 | 24.8004 | 9 | 100 | 6.378885 |
| Jordan | Abu Hamid | Chalcolithic | -4400 | 4 | 10.1 | 8.2 | 10.8 | *1.2629* | 4.784766 | 3 | 200 | 6.316347 |
| Jordan | Pella | Chalcolithic | -4300 | 34 | 9.42 | 6.5 | 12 | 1.476 | 71.89301 | 33 | 300 | 5.891088 |
| Israel | Nevallat | Chalcolithic | -3900 | 36 | 7.84 | *5.5* | *11.5* | 1.33 | 61.9115 | 35 | 700 | 4.902986 |
| Israel | Shoham | Chalcolithic | -3795 | 23 | 7.52 | 5 | 10 | 1.19 | 31.1542 | 22 | 805 | 4.702864 |
| Israel | Cave of the Treasure | Chalcolithic | -3500 | 58 | 9.35 | 5.82 | 12.36 | 1.34 | 102.3494 | 57 | 1100 | 5.846037 |
| Jordan | Pella | Early Bronze Age | -3150 | 5 | 9.50 | *7.20* | *10.50* | 1.14 | 5.1984 | 4 | 1450 | 5.941118 |
| Jordan | Tell esh Shuna | Early Bronze Age | -2600 | 70 | 9.83 | 7 | 13.3 | 1.324999 | 121.138 | 69 | 2000 | 6.1466 |
| Israel | Lachish | Early Bronze Age | -2500 | 20 | 11.2 | 9 | 13.2 | 1.124513 | 24.02606 | 19 | 2100 | 7.004266 |
| Jordan | Pella | LBA | -1325 | 41 | 10.72 | *8.4* | *13* | 0.999 | 39.92004 | 40 | 3275 | 6.704083 |
| Israel | Tel Jerisheh | LBA | -1300 | 28 | 10.71 | 9 | 12.5 | 0.97 | 25.4043 | 27 | 3300 | 6.697829 |
| Greece | Tiryns | LBA | -1250 | 25 | *10.05* | 5.1 | 15 | 2.51868 | 152.25 | 24 | 3350 | 6.285078 |
| Israel | Tell Keisan | Iron Age | -1000 | 100 | 11.31 | 8.33 | 13.33 | 0.996971 | 98.40121 | 99 | 3600 | 7.073057 |
| Jordan | Pella | Iron Age | -950 | 171 | 10.78 | *7.4* | *13* | 1.095 | 203.8343 | 170 | 3650 | 6.741606 |
| Israel | Lachish | Iron Age | -500 | 25 | 10.1 | 8.2 | 12.6 | 1.119413 | 30.07407 | 24 | 4100 | 6.316347 |
| Israel | Gamla | Hellenistic | -150 | 64 | 12.85 | 10 | 14.5 | 1.19 | 89.2143 | 63 | 4450 | 8.036144 |
|  |  |  |  |  | 824 |  |  |  | sums | 1288.815 | 806 |  |
|  |  |  |  |  |  |  |  |  | **σ (s sum/ n sum)** | **1.599026** |  |  |

**Fig. 6** Graph of Haldane rate estimate for *Olea europea,* based on a generation time of 10 years

**Table 7** Metrical data on avocado (*Persea americana*) seeds. Measurements from C. Smith 1966; 1969, with ages re-estimated on the basis of B. Smith 2005

| Region | Site | Period | Strati-graphic phase | Preserv. | Median date estimate | N | L. ave | L. min | L. max | stdev | W. ave | W. min | W. max | stdev | source |
| --- | --- | --- | --- | --- | --- | --- | --- | --- | --- | --- | --- | --- | --- | --- | --- |
| Tehuacan | Coxcatlan Cave | Abjas and Coxcatlan | pre-ceramic | charred | -3000 | 4 | 1.95 | 1.8 | 2.1 | 0.1291 | 1.7 | 1.4 | 2.1 | 0.2944 | Smith 1966 |
| Tehuacan | Coxcatlan Cave | Santa Maria | TC 50 VII | charred | -400 | 14 | 2.2071 | 1.4 | 2.8 | 0.3812 | 2 | 1.2 | 2.8 | 0.4788 | Smith 1966 |
| Tehuacan | El Riego Cave | Santa Maria | TC 35e E | charred | -300 | 2 | 2.3 | 1.6 | 3 | 0.9900 | 1.75 | 1.6 | 1.9 | 0.2121 | Smith 1966 |
| Tehuacan | Coxcatlan Cave | Palo Blanco | TC 50 IV-V | charred | 250 | 19 | 2.0053 | 1.3 | 3.4 | 0.5254 | 1.93 | 1.5 | 3 | 0.4056 | Smith 1966 |
| Tehuacan | Coxcatlan Cave | Palo Blanco | TC 50 VI | charred | -50 | 64 | 2.0984 | 1.5 | 3.2 | 0.3786 | 1.8 | 1.1 | 3.5 | 0.6034 | Smith 1966 |
| Tehuacan | Purron Cave | Palo Blanco (includes Snta Maria) | TC 272 D | charred | 200 | 7 | 2.3857 | 1.9 | 3.1 | 0.4180 | 1.885714 | 1.4 | 3 | 0.5900 | Smith 1966 |
| Tehuacan | El Riego Cave | Palo Blanco | TC 35e D | charred | 250 | 4 | 2.625 | 2.2 | 2.9 | 0.3100 | 1.775 | 1.5 | 2.1 | 0.2500 | Smith 1966 |
| Tehuacan | El Riego Cave | Palo Blanco | TC 35e C | charred | 600 | 6 | 2.5167 | 1.8 | 3.5 | 0.5776 | 1.766667 | 1.4 | 2 | 0.2582 | Smith 1966 |
| Oaxaca | Guila Naquitz | Monte Alban IIIb-IV | OC-43 Zone A | -10% (desicc. | 700 | 118 | 2.385 | 1.53 | 2.97 | 0.2806 | 1.845 | 1.35 | 2.25 | 0.1754 | Smith 1969 |
| Tehuacan | Coxcatlan Cave | Venta Salada | TC 50 III-I | charred | 1200 | 16 | 2.3875 | 1.7 | 3.8 | 0.6281 | 2.075 | 1.3 | 3.1 | 0.4768 | Smith 1966 |
| Tehuacan | El Riego Cave | Venta Salada | TC 35e B | charred | 1300 | 6 | 2.5333 | 1.8 | 3 | 0.4457 | 1.783333 | 1.2 | 2.4 | 0.5154 | Smith 1966 |
| Tehuacan | El Riego Cave | Venta Salada | TC 35e A | charred | 1450 | 4 | 2.65 | 1.7 | 3.3 | 0.6807 | 2.05 | 1.5 | 2.5 | 0.4123 | Smith 1966 |

Mann-Kendall trend test of ave.L. all *Persea americana, p*(no trend): 0.003192*

Mann-Kendall trend test of ave.L. *Persea americana* 400 BC-1450AD*, p*(no trend): 0.012731*

**Table 8** *Persea americana* domestication episode data for haldanes calculation

| **Site** | **Period** | **Median Age** | **n** | **L. ave** | **L. stdev** | **s-terms (stdev^2^x (n-1))** | **n-terms (n-1)** | **Gener-ations (10-yr)** | **x/σ** |
| --- | --- | --- | --- | --- | --- | --- | --- | --- | --- |
| Coxcatlan Cave | Santa Maria | -400 | 14 | 2.207143 | 0.381221 | 2.98 | 13 | 10 | 13.60965 |
| El Riego Cave | Santa Maria | -300 | 2 | 2.3 | 0.989949 | 0.045 | 1 | 20 | 14.18222 |
| Coxcatlan Cave | Palo Blanco | 250 | 19 | 2.005263 | 0.525435 | 2.961053 | 18 | 75 | 12.36482 |
| Coxcatlan Cave | Palo Blanco | -50 | 64 | 2.098438 | 0.378591 | 22.94 | 63 | 45 | 12.93935 |
| Purron Cave | Palo Blanco (includes Snta Maria) | 200 | 7 | 2.385714 | 0.418045 | 2.088571 | 6 | 70 | 14.71075 |
| El Riego Cave | Palo Blanco | 250 | 4 | 2.625 | 0.30957 | 0.1875 | 3 | 75 | 16.18623 |
| El Riego Cave | Palo Blanco | 600 | 6 | 2.516667 | 0.577639 | 0.333333 | 5 | 110 | 15.51823 |
| Guila Naquitz | Monte Alban IIIb-IV | 700 | 118 | 2.385 | 0.280571 | 3.597755 | 117 | 120 | 14.70635 |
| Coxcatlan Cave | Venta Salada | 1200 | 16 | 2.3875 | 0.628092 | 3.41 | 15 | 170 | 14.72176 |
| El Riego Cave | Venta Salada | 1300 | 6 | 2.533333 | 0.44572 | 1.328333 | 5 | 180 | 15.621 |
| El Riego Cave | Venta Salada | 1450 | 4 | 2.65 | 0.680686 | 0.51 | 3 | 195 | 16.34038 |
|  |  |  |  |  | Sums | 40.38155 | 249 |  |  |
|  |  |  |  |  | σ (s sum/ n sum) | 0.162175 |  |  |  |

**Fig. 7** Graph of Haldane rate estimate for *Persea americana,* based on a generation time of 10 years

**Table 9** *Castanea crenata* metrical data. Yoshikawa (2011) provided raw measurements, while Nishiro and Sasaki (2014) provided derived calculations of nut mass index (square root of length x width) for a larger number of sites

| Site | Period | age | n | L. ave | stdev | W.ave | stdev | n | mass index √(HxW) | Stdev |
| --- | --- | --- | --- | --- | --- | --- | --- | --- | --- | --- |
| Awazu Kotei | Initial Jomon | -7425 |  |  |  |  |  | 8 | *16* | *2.10733* |
| Torihama | Early Jomon | -3800 |  |  |  |  |  | 17 | 22 | 2.787161 |
| Sannai Maruyama | Early Jomon | -4050 | 15 | 19 | 4.9 | 24.5 | 7.4 | 46 | 22 | 3 |
| Ofune C | Middle Jomon | -3000 |  |  |  |  |  | 84 | 20 | 3 |
| Wadai | Middle Jomon | -2500 |  |  |  |  |  | 18 | 27 | 3 |
| Benten-ike | Late Jomon | -1900 | 6 | 25.3 | 4.2 | 25.1 | 3.4 | 6 | 25 | 4 |
| Noji | Final Jomon | -955 | 33 | 28.9 | 4.7 | 29.1 | 5.9 |  |  |  |
| Yachi | Final Jomon | -900 |  |  |  |  |  | 33 | 28 | 5 |
| Yonaizumi | Final Jomon | -945 | 8 | 29.6 | 3.2 | 31.7 | 3.6 | 8 | 31 | 3 |
| Aota | Final Jomon | -700 | 46 | 30.4 | 6.1 | 33.5 | 8.3 | 63 | 31 | 6 |
| Shimokanomizu | Final Jomon | -1000 |  |  |  |  |  | 85 | 22 | 3 |

Mann-Kendall trend test of nut mass index. all *Castanea crenta, p*(no trend): 0.0046*

**Table 10** *Castanea crenata* domestication episode data for haldanes calculation based on nut mass index

| **Site** | **Period** | **N** | **Median Age** | **mass index √(HxW)** | **stdev** | **s-terms (stdev^2^x (n-1))** | **n-terms (n-1)** | **Gener-ations (10-yr)** | **x/σ** |
| --- | --- | --- | --- | --- | --- | --- | --- | --- | --- |
| Ofune C | Middle Jomon | 84 | -3000 | 20 | 3 | 747 | 83 | 0 | 1.011043 |
| Wadai | Middle Jomon | 18 | -2500 | 27 | 3 | 153 | 17 | 50 | 1.364908 |
| Benten-ike | Late Jomon | 6 | -1900 | 25 | 4 | 80 | 5 | 110 | 1.263804 |
| Yachi | Final Jomon | 33 | -900 | 28 | 5 | 800 | 32 | 210 | 1.41546 |
| Yonaizumi | Final Jomon | 8 | -945 | 31 | 3 | 63 | 7 | 205.5 | 1.567117 |
| Aota | Final Jomon | 63 | -700 | 31 | 6 | 2232 | 62 | 230 | 1.567117 |
|  |  |  |  |  | Sums | 4075 | 206 |  |  |
|  |  |  |  |  | σ (s sum/ n sum) | 19.78155 |  |  |  |


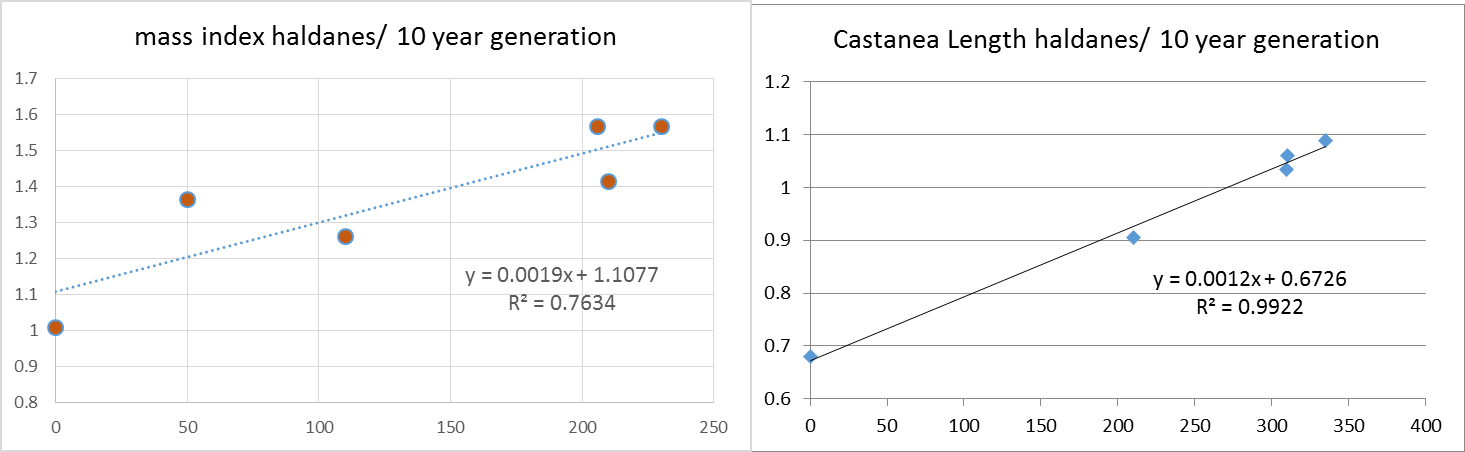


**Fig. 8** Graphs of haldane rate estimates for *Castanea crenata* using nut mass index (left) and fruit length (right), based on a generation time of 10 years

**Table 11** Metrical data and Haldane calculations for *Canarium* sp. from Talepakamalai (based on Lepofsky et al 1998)

|  |  |  |  |  |  |  | **Max diameter (mc)** | | |  | |  | | **s-terms** | | **n-terms** | | **Generation (5-year)** | | **x/σ** | |
| --- | --- | --- | --- | --- | --- | --- | --- | --- | --- | --- | --- | --- | --- | --- | --- | --- | --- | --- | --- | --- | --- |
| **Per.** | **Age** | **n** | **L. ave** | **min** | **max** | **stdev** | **D.ave** | **min** | **max** | | **stdev** | |  | |  | |  | |  | |  |
| Early | -1500 | 107 | 3.8 | 2.2 | 5 | 0.5 | 2.1 | 1.3 | 2.7 | | 0.2 | | 4.24 | | 106 | | 0 | | 93.74005 | |  |
| Mid. | -1000 | 79 | 4 | 3 | 5 | 0.4 | 2.1 | 1.3 | 2.7 | | 0.2 | | 3.12 | | 78 | | 100 | | 98.67374 | |  |
| Late | -700 | 3 | 4 | 3.5 | 4.4 | 0.5 | 2 | 1.7 | 2.2 | | 0.3 | | 0.18 | | 2 | | 160 | | 98.67374 | |  |
|  |  |  |  |  |  |  |  |  |  | | Sums | | 7.54 | | 186 | |  | |  | |  |
|  |  |  |  |  |  |  |  |  |  | | σ (s sum/ n sum) | | 0.040538 | |  | |  | |  | |  |

Mann-Kendall trend test of ave.L. all *Canarium,* including modern reference material reported by Lepofksy et al. 1998, *p*(no trend): 0.042*

**Table 12** Metrical data and Haldane calculations for *Spondias* sp. from Talepakamalai (based on Lepofsky et al 1998)

| **Period** | **Age** | **n** | **L. ave** | **min** | **max** | **stdev** | **D.ave** | **min** | **max** | **stdev** | **s-terms** | **n-terms** | **Generation (5-year)** | **x/σ** |
| --- | --- | --- | --- | --- | --- | --- | --- | --- | --- | --- | --- | --- | --- | --- |
| Early | -1500 | 39 | 2.1 | 1 | 3 | 0.4 | 2 | 1.4 | 2.8 | 0.3 | 5.67 | 63 | 0 | 26.85279 |
| Middle | -1000 | 64 | 2.3 | 1.5 | 3.3 | 0.3 | 2.1 | 1.1 | 2.7 | 0.3 | 1.89 | 21 | 100 | 29.18782 |
| Late | -700 | 22 | 2.5 | 1.9 | 3 | 0.3 | 2.3 | 1.8 | 3.2 | 0.3 | 0.32 | 8 | 160 | 30.35533 |
|  |  |  |  |  |  |  |  |  |  | Sums | 7.88 | 92 |  |  |
|  |  |  |  |  |  |  |  |  |  | σ (s sum/ n sum) | 0.085652 |  |  |  |

Mann-Kendall trend test of ave.L. all *Spondias,* including modern reference material reported by Lepofksy et al. 1998, *p*(no trend): 0.042*

**References cited in supplementary material**

Beech M (2003) Archaeobotanical evidence for early date consumption in the Arabian Gulf. In: Al-Swaidi JS (ed) The date palm: from traditional resource to green wealth. The Emirates Center for Strategic Studies and Research, Abu Dhabi, pp 11-31

Bookidis N, Hansen J, Snyder L, Goldberg P (1999) Dining in the Sanctuary of Demeter and Kore at Corinth. Hesperia: The Journal of the American School of Classical Studies at Athens 68:1-54

Clapham AJ, Stevens CJ (2009) Dates and confused: does measuring date stones make any sense? In: Ikram S, Dodson A (eds) Beyond the Horizon: Studies in Egyptian Art, Archaeology and History in Honour of Barry J. Kemp*.* Supreme Council of Antiquities, Cairo, pp 9-27

Costantini L (1985) Considerazioni su alcuni reperti di palma da dattero e sul centro di origine e l’area di coltivazione della Phoenix dactylifera L. In: Gnoli G, Lanciotti L (eds) Orientalia Josephi Tucci memoriae dicata. Serie Orientale 56. Istituto Italiano per il Medio ed Estremo Oriente, Rome, pp 209-218 (in Italian)

Costantini L, Audisio P (2000) Plant and insect remains from the Bronze Age site of Ra’s al-Jinz (RJ-2), Sultanate of Oman. Paleorient 26(1): 143-156

Dighton A, Fairbairn A, Bourke S, Faith JT, Habgood P (2017) Bronze Age olive domestication in the north Jordan valley: new morphological evidence for regional complexity in early arboricultural practice from Pella in Jordan. Veget Hist Archaeobot 26:403-413

Fuller DQ (2000) The Botanical Remains. In: Insoll T (ed) Urbanism, Archaeology and Trade. Further Observations on the Gao Region (Mali): The 1996 Fieldseason Results. BAR S829. Archaeopress, Oxford, pp 28-35

Helbaek H (1958) Plant Economy in Ancient Lachish. In: Tufnell O (ed) Lachish IV: The Bronze Age. Oxford University Press, Oxford, pp 309-317

Kislev ME (1995) Wild Olive Stones at Submerged Chalcolithic Kfar Samir, Haifa, Israel. J Israel Prehist Soc 26:134-145

Lepofsky D, Kirch PV, Lertzman KP (1998) Metric analyses of prehistoric morphological change in cultivated fruits and nuts: an example from Island Melanesia. J Archaeol Sci 25:1,001-1,014

Liphschitz N, Bonani, G (2000) Dimensions of Olive (*Olea europaea*) stones as a reliable parameter to distinguish between wild and cultivated varieties: further evidence. Tel Aviv 27:23-25

Liphschitz N, Bonani G (2001) Wild and Cultivated Date Palm (*Phoenix dactylifera*) from Qumran Cave 24. Tel Aviv 28:305-309

Liphschitz N, Gophna R, Bonani G, Feldstein A (2013) Wild olive (*Olea europaea*) stones from a chalcolithic cave at shoham, Israel and their implications. Tel Aviv 23:135-142

Lone FA, Khan M, Buth GM (1993). Palaeoethnobotany. Plants and ancient man in Kashmir. Oxford & IBH Publishing Co., New Delhi

Miller NF (1984) Some Plant Remains from Khirokitia, Cyprus: 1977 and 1978 Excavations. In : LeBrun A (ed) Fouilles récentes à Khirokitia (Chypre) 1977-1981. Mémoire 41. Editions Recherches sur les civilisations, Paris, pp 183-188

Nesbitt M (1993) Archaeobotanical Evidence for Early Dilmun Diet at Saar, Bahrein. Arabian Archaeology and Epigraphy 4:20-47

Nixon S, Murray MA, Fuller DQ (2011) Plant use at an early Islamic merchant town in the West African Sahel: the archaeobotany of Essouk-Tadmakka (Mali). Veget Hist Archaeobot 20:223-39

Noshiro S, Sasaki Y (2014) Pre-agricultural management of plant resources during the Jomon period in Japan—a sophisticated subsistence system on plant resources. J Archaeol Sci 42:93-106

Rowley-Conwy P (1987) Remains of date (*Phoenix dactylifera*) from Failaka, Kuwait. In: Højlund F (ed) Danish archaeological investigations on Failaka, Kuwait. The second millenium settlements. Jutland Archaeological Society Publications, pp 181-183

Runnels CN, Hansen J (1986) The olive in the prehistoric Aegean: the evidence for domestication in the early Bronze Age. Oxford J Archaeol 5:299-308

Sadori L, Allevato E, Bosi G, Caneva G, Castiglioni E, Celant A, Di Pasquale G, Giardini M, Mazzanti M, Rinaldi R, Rottoli M (2009) The introduction and diffusion of peach in ancient Italy. In: Morel, J-P, Mercuri AM (eds) Plants and culture: seeds of the cultural heritage of Europe. Edipuglia, Bari, pp 45-61

Samuel D (2001) Archaeobotanical evidence and analysis. In: Berthier S (ed) Peuplement rural et amenagements hydroagricoles dans la moyenne vallee de l'Euphrate fin VIIe-XIXe siecle. Institut Français d'Etudes Arabes de Damas, Damascus, pp.343-481

Smith BC (2005) Reassessing Coaxcatlan Cave and the early history of domesticated plants in Mesoamerica. PNAS 102:9,438-9,445

Smith CEJ (1966) Archaeological evidence for selection in avocado. Econ Bot 20:169-175

Smith CEJ (1969) Additional notes on pre-conquest avocados in Mexico. Econ Bot 23:135-140

Smith W (2003) Archaeobotanical Investigations of Agriculture at Late Antique Kom el-Nana (Tell el-Amarna). Egypt Exploration Society, London

Van den Brink EC, Liphschitz N, Lazar D, Bonani G (2001) Chalcolithic dwelling remains, cup marks and olive (*Olea europaea*) stones at Nevallat. Israel Exploration Journal 51:36-43

Van der Veen M (2011) Consumption, trade and innovation. Africa Magna Verlag, Frankfurt

Willcox G (1977) Exotic plants from Roman waterlogged sites in London. J Archaeol Sci 4:269-282

Yoshikawa M (2011) Dispersal of *Castanea crenata* pollen and distribution of *C. crenata* forest around the Sannai-maruyama site during the Jomon period. Jpn J Hist Bot 18:65-76 (in Japanese)

Zaitschek DV (1980). Plant remains from the cave of the treasure. In: Bar-Adon P (ed) The cave of the treasure. Jerusalem, pp 223-227

Zheng Y, Crawford GW, Chen X (2014) Archaeological evidence for peach (*Prunus persica*) cultivation and domestication in China. PloS one 9:e106595
